# The Tumor-Suppressive *miR-497-195* Cluster Targets Multiple Cell-Cycle Regulators in Hepatocellular Carcinoma

**DOI:** 10.1371/journal.pone.0060155

**Published:** 2013-03-27

**Authors:** Mayuko Furuta, Ken-ichi Kozaki, Kousuke Tanimoto, Shinji Tanaka, Shigeki Arii, Teppei Shimamura, Atsushi Niida, Satoru Miyano, Johji Inazawa

**Affiliations:** 1 Department of Molecular Cytogenetics, Medical Research Institute and School of Biomedical Science, Graduate School of Medicine, Tokyo Medical and Dental University, Tokyo, Japan; 2 Global Center of Excellence (GCOE) Program for International Research Center for Molecular Science in Tooth and Bone Diseases, Graduate School of Medicine, Tokyo Medical and Dental University, Tokyo, Japan; 3 Department of Therapeutic Genomics, Graduate School of Medicine, Tokyo Medical and Dental University, Tokyo, Japan; 4 Hard Tissue Genome Research Center, Graduate School of Medicine, Tokyo Medical and Dental University, Tokyo, Japan; 5 Genome Laboratory, Graduate School of Medicine, Tokyo Medical and Dental University, Tokyo, Japan; 6 Department of Hepato-Biliary-Pancreatic Surgery, Graduate School of Medicine, Tokyo Medical and Dental University, Tokyo, Japan; 7 Research Fellow of the Japan Society for the Promotion of Science, Institute of Medical Science, University of Tokyo, Tokyo, Japan; 8 Human Genome Center, Institute of Medical Science, University of Tokyo, Tokyo, Japan; Virginia Commonwealth University, United States of America

## Abstract

MicroRNAs (miRNAs) are key post-transcriptional regulators of gene expression and commonly deregulated in carcinogenesis. To explore functionally crucial tumor-suppressive (TS)-miRNAs in hepatocellular carcinoma (HCC), we performed integrative function- and expression-based screenings of TS-miRNAs in six HCC cell lines. The screenings identified seven miRNAs, which showed growth-suppressive activities through the overexpression of each miRNA and were endogenously downregulated in HCC cell lines. Further expression analyses using a large panel of HCC cell lines and primary tumors demonstrated four miRNAs, *miR-101*, *-195*, -*378* and *-497*, as candidate TS-miRNAs frequently silenced in HCCs. Among them, two clustered miRNAs *miR-195* and *miR-497* showed significant growth-suppressive activity with induction of G1 arrest. Comprehensive exploration of their targets using Argonute2-immunoprecipitation-deep-sequencing (Ago2-IP-seq) and genome-wide expression profiling after their overexpression followed by pathway analysis, revealed a significant enrichment of cell cycle regulators. Among the candidates, we successfully identified *CCNE1*, *CDC25A*, *CCND3*, *CDK4*, and *BTRC* as direct targets for *miR-497* and *miR-195*. Moreover, target genes frequently upregulated in HCC in a tumor-specific manner, such as *CDK6*, *CCNE1*, *CDC25A* and *CDK4*, showed an inverse correlation in the expression of *miR-195* and *miR-497*, and their targets. These results suggest the molecular pathway regulating cell cycle progression to be integrally altered by downregulation of *miR-195* and *miR-497* expression, leading to the aberrant cell proliferation in hepatocarcinogenesis.

## Introduction

HCC, the most common primary liver cancer, is an extremely lethal disease, which causes about 700,000 deaths worldwide annually [1]. As proposed by Vogelstein and Kinzler [2] in overall cancer, hepatocarcinogenesis is a DNA disease due to the accumulation of altered genes that control the cell cycle and cell proliferation, and a large number of genetic and epigenetic alterations accumulate during this process. Since master regulators of the cell cycle are also indispensable for normal cells, current anti-cancer therapeutic strategies have shifted to the search for one single dominant oncogene or addictive molecule that only tumors rely on, and proposed as a concept for oncogene addiction [3]–[6]. However, successful translation of the oncogene addiction model into the rational and effective design of targeted therapeutics against individual oncoproteins still faces major obstacles, mainly due to the emergence of escape mechanisms, drug resistance and basically tumor-individuality arising from “off-label patients” as termed by Torti and Trusolino [7]. Recently, an increasing number of reports have described a new class of small regulatory RNA molecules termed microRNAs (miRNAs) implicated in hepatocarcinogenesis, and seems to open the possibility of raising new therapeutics mimicking endogenous miRNA machineries [8].

miRNAs are endogenous small non-coding RNAs which act as negative regulators for mRNA expression via sequence-complementary targeting of the 3′ untranslated region (3′UTR) to repress translation or mediate mRNA degradation [9]. Due to their abundance and divergence of targeting specificity, it is believed that one single miRNA can interact with multiple mRNA targets [10] to achieve regulatory control over virtually every biological process [11]. Although hundreds of miRNAs are known to have deregulated expression in cancer with accumulating evidence demonstrating that miRNAs have oncogenic or tumor-suppressive (TS) functions [12], molecular pathway underlying these miRNAs are poorly understood. Therefore, identifying the cluster of target genes for a cancer-related miRNA is essential to provide them as a promising therapeutic agent.

In the study presented here, we first explored the promising TS-miRNAs for HCC by screenings based on their expression status and growth-suppressive activity in HCC cells. We also tested an integrative approach to identify a set of target genes for these TS-miRNAs that explain the whole picture of their function in HCCs.

## Results

### A combination of function- and Expression-based Screenings Identified Putative TS-miRNAs in HCC Cells

**Figure 1:**
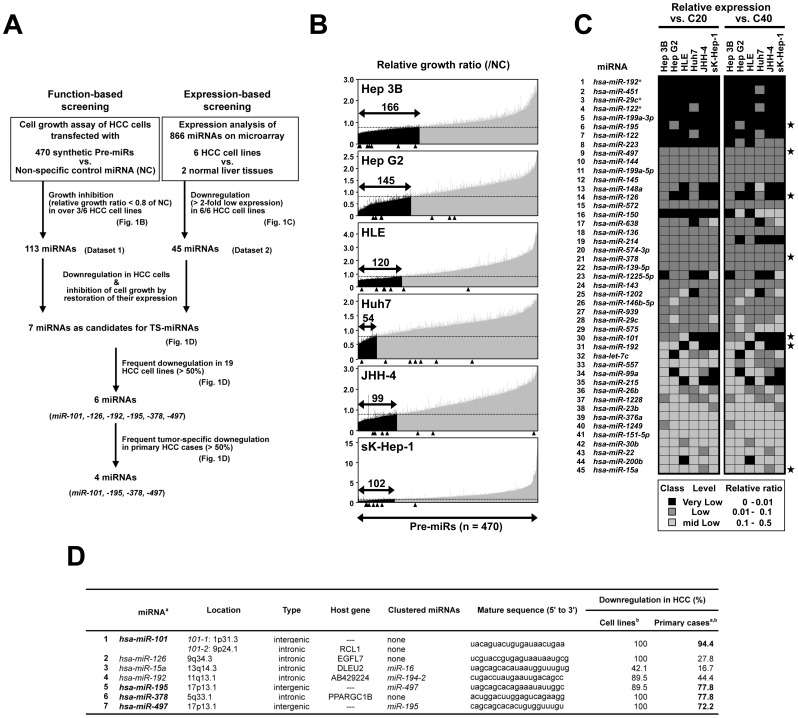
An integrative approach to the identification of TS-miRNAs using function- and expression-based screening in HCC cell lines. **A**, Strategy for the identification of TS- miRNAs by function- and expression-based screening in 6 HCC cell lines. **B**, Results of function-based screening in 6 HCC cell lines. The numbers of viable cells 4–5 days after transfection with 5 nM of 470 dsRNAs mimicking mature miRNAs loaded on Pre-miR™ miRNA Precursor Library-Human V3 (Ambion) or control non-specific dsRNA were evaluated by the WST-8 assay in duplicate. Relative *in vitro* cell growth ratios in these cell lines were calculated by normalization of each result to the cell numbers in control cells transfected with non-specific miRNA (see Dataset S1). The lower solid arrow indicates the 470 miRNAs examined. Closed arrows in each graph indicate candidate miRNAs with marked growth inhibitory effects (growth ratio <0.8) compared with the control counterpart in our function-based screening. The arrowhead indicates the results for seven candidate miRNAs conclusively selected in the function- and expression-based screening (see Fig. 1D). **C**, Summary of expression profiles of 45 candidate miRNAs, expression levels of which were downregulated (>2-fold) in all 6 cell lines as compared with those of two normal liver tissues (C20 and C40, see Dataset S2). Stars indicate the results of the same seven candidate miRNAs as in Fig. 1B. **D**, Seven candidates for tumor-suppressive miRNAs involved in HCC pathogenesis identified through function- and expression-based screening approach. MiRNAs frequently downregulated (>50%) in tumors compared with their paired non-tumorous liver tissues in clinical HCC cases are in bold face (^a^). Expression level of mature form for each miRNA was evaluated inTaqMan® MicroRNA Assays (Applied Biosystems) as described in Materials and Methods (^b^).

To identify TS-miRNAs, we first performed an integrative approach using function- and expression-based screenings in six HCC cell lines (Hep G2, Hep 3B, HLE, Huh7, JHH-4, and sK-Hep-1). We focused on miRNAs, which showed remarkable inhibition of cell proliferation *in vitro* together with significant downregulation in HCC cell lines compared with normal liver tissues (Fig. 1A), as candidate TS-miRNAs for HCC.

In the function-based screening in six HCC cell lines using a synthetic miRNA mimic library containing 470 pre-miRNAs, 113 miRNAs demonstrated remarkable inhibitory effects on cell growth in more than 3 of 6 cell lines (relative growth ratio <0.8 compared with control non-specific miRNA; Fig. 1B, and Dataset S1).

In the expression-based screening in the same six cell lines and normal liver tissues (C20, C40) using a miRNA microarray containing 866 human miRNAs, 265 miRNAs were able to be quantified at least in one cell line. Among them, 194, 194, 77, 190, 199, and 168 miRNAs were downregulated (>2-fold decrease) in the Hep G2, Hep 3B, HLE, Huh7, JHH-4, and sK-Hep-1 cell lines, respectively, compared with C20 and C40, and 45 miRNAs were commonly downregulated in all these six cell lines (>2-fold decrease; Fig. 1C, and Dataset S2).

By combining results of the two screenings, we identified seven miRNAs, *i.e. miR-101*, *-126*, *-15a*, *-192*, *-195*, *-378* and *-497*, as candidate TS-miRNAs for HCC (Fig. 1A and 1D). Among them, *miR-101* and *miR-195* had already been reported as possible TS-miRNAs for HCC [13], [14], suggesting that the approach employed in this study could successfully identify TS-miRNAs in HCC cells.

### *miR-195* and *miR-497* Emerged as Possible TS-miRNAs

In order to narrow down those seven candidates by frequency of their downregulation in HCC cells, we next performed expression analyses in a panel of 19 HCC cell lines (Fig. S1A) and a panel of paired tumorous and non-tumorous tissues from 18 primary HCC cases (Fig. S1B). *miR-15a* was excluded due to a low frequency of downregulation in a panel of HCC cell lines (42.1%) and primary cases (16.7%, Fig. 1D). *miR-126* and *miR-192* were also excluded due to a low frequency of tumor-specific down-regulation in a panel of primary HCC cases (27.8% and 44.4%, respectively; Fig. 1D). Finally, four miRNAs, *miR-101*, *miR-195*, *miR-378*, and *miR-497*, were selected as the most promising candidates for TS-miRNA for HCC showing frequent (>50%) tumor-specific downregulation both in HCC cell lines and primary HCC cases (Fig. 1D). Among them, *miR-101* was excluded from further analysis because of its well-known function as a TS-miRNA in HCC [13]. Notably, *miR-378* had been reported as an oncomiR in various types of cancers [15], [16], while showing TS function in our screening performed in HCC. In addition, *miR-378* was also reported to be downregulated during liver regeneration after partial hepatectomy in mice with a delay in cell cycle progression involving the G1 to S phase transition [17], suggesting *miR-378* to have cell growth suppressive activity in liver and supporting our findings in HCCs. These inconsistent findings indicate that miR-378 may have multiple functions in a tissue- or lineage-specific manner. Since we focused on miRNAs showing direct inhibition of HCC cell growth in this screening, we excluded miR-378 from further analysis. Among the 4 candidates, *miR-497* and *miR-195* belong to the same miRNA family and are also located very close together: pre-*miR-195* is 209 bp downstream from the 3′ end of pre-*miR-497* at 17p13.1, and is frequently deleted in human cancers [18], [19]. We focused on these two miRNAs for further analysis, because *(a)* reported evidence and evolutional conservation suggest that the clustered miRNAs may have related physiological and pathophysiological functions including carcinogenesis [20]–[22], and *(b) miR-497* is poorly characterized in human cancer, especially in HCC, indeed, few target molecules for those two miRNAs are known, although several reports have suggested *miR-195* to be a possible TS-miRNA for various human cancers including HCC [14], [23]–[25].

### Mechanisms Underlying Deregulation of *miR-195* and *miR-497* Expression in HCC

Computational analysis using a data set of miRNA expression profiles in 89 primary HCV-related HCCs obtained from the database of the Broad Institute (GSE20596) revealed a significantly positive correlation between expression levels of mature forms of *miR-195* and *miR-497* (Fig. S2A, left panel). In addition, the expression status of *miR-195* and *miR-497* seemed to be correlated regardless of their viral infection status not only in tumor samples but also in non-tumorous liver tissues, when we recalculated the data used in Fig. S1B (Fig. S2A right panel), although the number of samples are too small for statistical analysis. These results suggesting those miRNAs to be transcribed concomitantly as a single primary transcript and expressed as a cluster not only in HCCs but also in non-tumorous liver tissues. Indeed, this hypothesis is supported by the existence of the transcript NR_038310.1, mir-497-195 cluster host gene (non-protein coding) (MIR497HG), and non-coding RNA in the NCBI reference sequence (http://www.ncbi.nlm.nih.gov/nuccore/NR_038310?report=GenBank).

We first examined copy-number aberrations around *miR-195* and *miR-497* in 18 HCC cell lines by q-gPCR, and found that only 3 of them (HLE, JHH-6, PLC/PRF/5) have a copy number loss at this locus in spite of frequent downregulation of those miRNAs in a number of HCC cell lines (Fig. S2B). Since the biogenesis of miRNAs has been reported to be altered in cancer cells [26], we tried to test the possibility that the down-regulation of those miRNAs were caused by abnormal miRNA biogenesis. Since downregulation of miR-195 and miR-497 was also observed in pri-miRNA level, those miRNAs seem to be downregulated at a transcriptional level rather than a post-transcriptional level (Fig. S2C). To assess other factors contributing to the downregulation of *miR-195* and *miR-497*, we next searched for a regulatory element for the putative polycistronic primary transcript, pri-*miR-497-195*. The putative transcriptional start site (TSS) was detected 1.6 kb upstream of pre-*miR-497* by SwitchGear Genomics Transcription Start Sites (CHR17_M0118_R1) and ENCODE Transcription Factor ChIP-seq (TAF1) binding site (UCSC Genome Bioinfomatics, Feb. 2009 [GRCh37/hg19] assembly; http://genome.ucsc.edu/cgi-bin/hgGateway) (Fig. S3A). Promoter activities around the TSS were confirmed in three HCC cell lines, Hep G2, sK-Hep-1 and HuH-6, regardless of the expression status of *miR-195* and *miR-497* (Fig. S3B). Although Suzuki et al. [27] reported that *miR-497* and *miR-195* are possibly regulated by non-CpG island methylation in colorectal cancer based on data from a genome-wide combined analysis of mRNA expression, chromatin signature, and DNA methylation, only minimal restoration was observed in the expression of those miRNAs after treatment with 5-aza-dCyd in HCC cell lines (Fig. S3C) and almost no difference in DNA methylation pattern around the TSS of pri-*miR-497-195* was observed among HCC lines regardless of their expression status (Fig. S3D). Moreover, the inhibition of the class I and II histone deacetylase (HDAC) families with Tricostatin A (TSA) did not induce restoration of *miR-195* nor *miR-497* in most of cell lines, suggesting that these HDACs do not strongly contribute to the decreased expression of those miRNAs in HCC cells (Fig. S3C). In addition, ChIP analyses around TSS for H3K4me3 and H3K27me3, which are histone modification patterns activating and inactivating gene expression, revealed that the promoter region of pri-*miR-497-195* showed no significant repressive pattern in the H3K27me3 modification, but a clear inactive pattern in the H3K4me3 modification in non-expressing HCC cells compared with *miR-195*-expressing HuH-6 cells (Fig. S3E). Taken together, frequent downregulation of *miR-497* and *miR-195* expression occurred not mainly by genomic loss, DNA hypermethylation or altered miRNA biogenesis, but at least partly through repressive histone modifications in the HCC cell lines examined, although HDAC inhibitor had little or no effect on the expression and the epigenetic factors contributing to the downregulation remain to be clarified.

### Restoration of *miR-195* or *miR-497* Suppresses Cell Proliferation and Cell Cycle Progression *in vitro*

**Figure 2:**
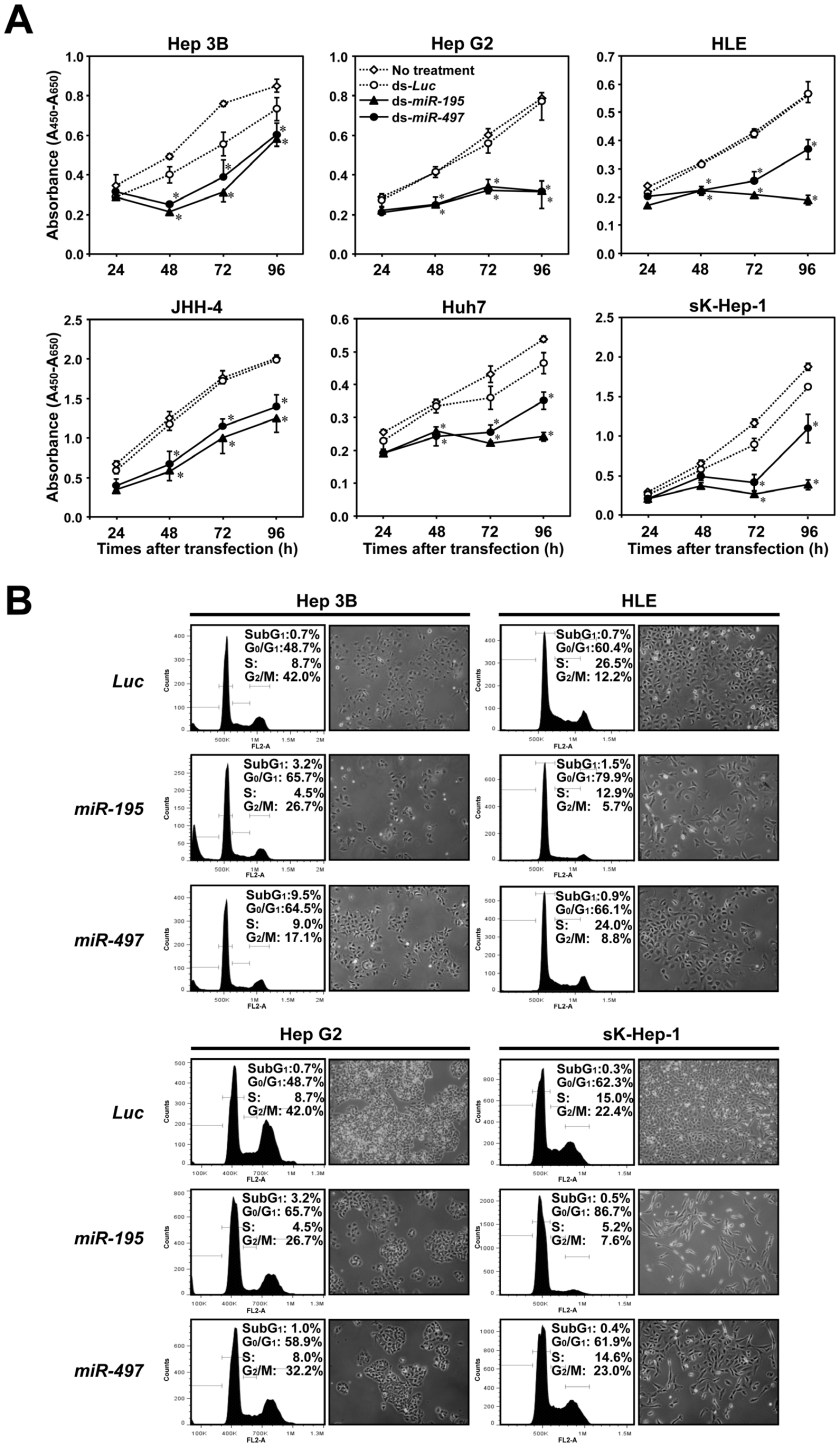
Growth suppressive effects of *miR-195* and *miR-497* on HCC cell lines lacking their expression. **A**, Growth curves of HCC cell lines after tansfection of 5nM of miCENTURY OX miNatural (Cosmo Bio) mimicking *miR-195* (black triangle) or *miR-497* (black circle), or control *Luc*, (white circle) assessed by WST-8 assay. Points, the mean of triplicate determinations in these experiments; bars, SD; Asterisks (*), *P*<0.05 versus *Luc* transfected cells in a statistical analysis with the Mann-Whitney *U* test. **B**, Results for the population in each phase of the cell cycle assessed by FACS (***left***) and phase-contrast micrographs (***right***) using HCC cell lines 72 hours after transfection of miCENTURY OX miNatural mimicking *miR-195* (***middle***) or *miR-497* (***lower***), or control *Luc* (***upper***).

Growth suppressive activities of double stranded RNAs (dsRNAs) mimicking *miR-195* and *miR-497* in HCC cell lines (Fig. 1B and Dataset S1) were validated by transfection of synthetic dsRNAs mimicking *miR-195* and *miR-497* and control non-specific miRNA purchased from a different company (Fig. 2A), excluding the off-target effects of synthetic dsRNAs. A similar growth suppression pattern for *miR-195* and *miR-497* was observed in 3 of 6 cell lines (Hep 3B, Hep G2 and JHH-4) by ectopic overexpression of those miRNAs, whereas comparatively weak growth suppression was observed after restoration of *miR-497* compared with *miR-195* in the remaining 3 cell lines (HLE, Huh7 and sK-Hep-1).

We next determined modes of action on the cell cycle by FACS analysis in several HCC cell lines (Fig. 2B). The accumulation in G0/G1 phase was significant in all cell lines transfected with *miR-195*, while the effect was slightly weak in HLE and sK-Hep-1 cells transfected with *miR-497* compared with *miR-195*. These findings were consistent with results of the cell growth assays *in vitro* (Fig. 2A). Although a small increase in cells in the sub-G1 fraction was observed in Hep 3B, no remarkable change in the number of cells was observed 48 hours after transfection (Fig. 2A), indicating G1 arrest to be predominant for the cell growth-suppressive effect of *miR-195* and *miR-497* in HCC cells. Taken together, *miR-195* and *miR-497* are likely to inhibit cell growth mainly by suppressing cell cycle progression, especially in the G1 to S phase.

### Identification of the Most Significant Biological Activities Regulated by *miR-195*/*miR-497* using GO *in silico*

For exploration of signaling pathways downstream of *miR-195* and *miR-497*, we performed a genome-wide mRNA expression analysis of Hep G2 and sK-Hep-1 cells 48 hours after transfection with *miR-195, miR-497*, or control dsRNA. Among significantly altered GO terms (correlated *p*-value<0.05) in biological processes and functions selected from genes showing 2-fold changes between *miR-195* or *miR-497* transfectants and their counterparts with a false discovery value (q) <0.001 (Table S1–1 to S–4), GO terms involved in the cell cycle most frequently appeared in all conditions. This result is consistent with the inhibitory effect of *miR-195* and *miR-497* on cell cycle (Fig. 2B), suggesting molecules contributing to cell cycle regulation to be major targets for *miR-195* and *miR-497*.

### Identification of Possible *miR-195* and/or *miR-497* Targets by Ago2-IP-deep Sequencing, Expression Analyses, and Bioinformatics Analyses

To determine direct targets for *miR-195* and/or *miR-497* responsible for hepatocaricinogenesis, we performed RNA coimmunoprecipitation with anti-Ago2 antibody (Ago2-IP) for trapping overexpressed miRNA incorporated into the RNA-induced silencing complex (RISC) together with their target mRNAs in Hep G2 cells transfected with *miR-195* or *miR-497*. Profiling of miRNAs obtained from the Ago2-IP fraction by miRNA microarray analysis showed a significantly increased rate of each transfected miRNA compared with most other endogenous miRNAs in Hep G2 cells (Fig. S4), indicating transfected miRNAs to be incorporated successfully and specifically into RISC. However, miRNAs other than *miR-195* or *-497* also showed increased rates, suggesting that mRNAs trapped by RISC include targets for non-specific miRNAs as false-positives. In this condition, we performed RNA sequencing using Ago2-IP RNAs (Ago2-IP-seq) along with total RNAs (mRNA-seq) from the same samples in *miR-195-*, *miR-497-*, or non-transfected Hep G2 cells, and calculated the abundance of each mRNA in the Ago2 binding fractions compared with total RNA fractions (Ago2-IP-seq/mRNA-seq of each RPKM value) in each sample (abundant-Ago2 fraction). Finally, we obtained the fold enrichment score by calculating the ratio of abundant-Ago2 fractions between *miR-195-* or *miR-497*-transfected samples and non-transfected samples.

In GSEA, genes selected as top 10% candidates by Ago2-IP in *miR-195* transfected cells were significantly enriched in genes downregulated by *miR-195* transfection, whereas those in *miR-497*-transfected cells were not significant (*p*<0.001 and = 0.0709607, FDR<0.001 and = 0.041126948, and normalized enrichment score = -1.7712895 and 1.1393404 for *miR-195* and *miR-497*, respectively; Fig. S4B). Through excluding genes without a target sequence of *miR-195* and *miR-497* in their 3′UTR, enrichment scores in GSEA were dramatically improved (*p*<0.001 and <0.001, FDR<0.001 and <0.001, and normalized enrichment score = -2.5568738 and -1.7213858 for *miR-195* and *miR-497*, respectively; Fig. S4C).

**Figure 3:**
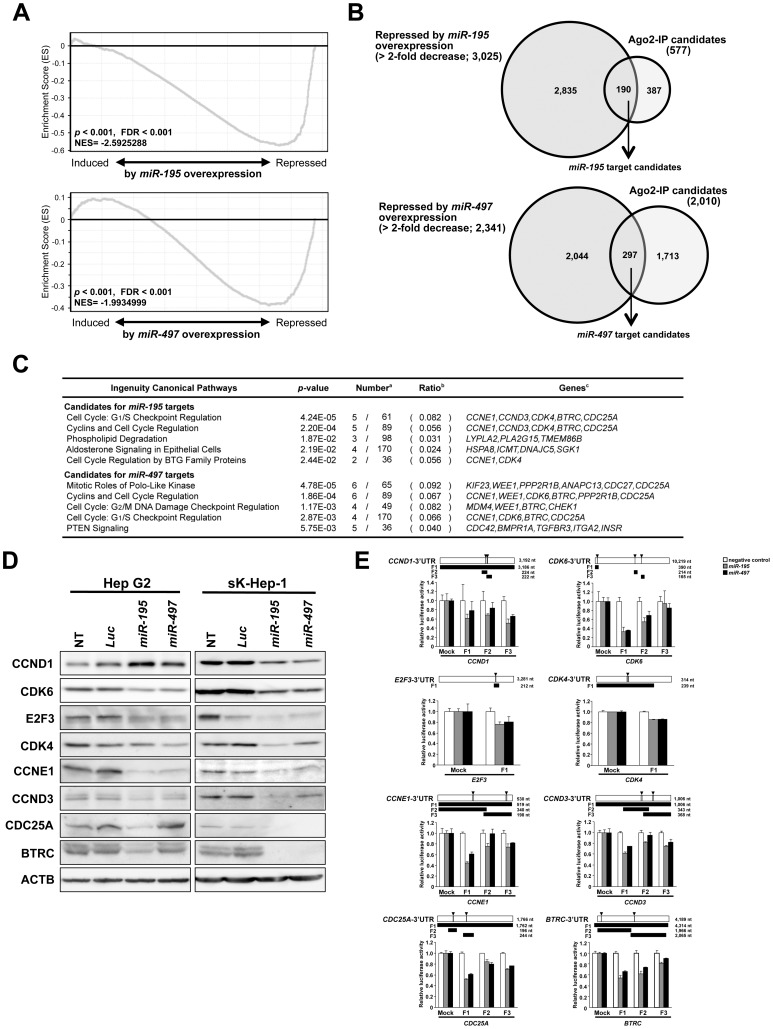
Exploration of possible direct *miR-195* and *miR-497* targets by Ago2-IP experiment. **A**, GSEA profiles of the Running Enrichment Score (ES) of genes enriched in the top 8% and 36% of candidates as target genes for *miR-195* (***upper***) and *miR-497* (***lower***) by Ago2-IP experiments along the rank of transcripts differentially expressed after transfection with *miR-195* (***left***) or *miR-497* (***right***) versus *Luc*. **B**, Outline for selecting final candidates as direct targets of *miR-195* (***upper***) and *miR-497* (***lower***) determined by a combination of Ago2-IP candidates and repressed genes by *miR-195* or *miR-497* (>2-fold decrease) in gene expression array experiments. **C,** Significantly enriched IPA canonical pathways for candidate direct target genes of *miR-195* and *miR-497*. Numbers(^a^), ratio(^b^) and genes (^c^) of candidate direct targets among all genes involved in the canonical pathway. **D**, Representative results of Western blotting of known targets, CDK6, CCND1, and E2F3, as positive controls and predicted targets, CDK4, CCNE1, CCND3, CDC25A, and BTRC, for *miR-195* and *miR-497*, 48 hours after transfection with miCENTURY OX miNatural mimicking *miR-195*, *miR-497*, or control *Luc*. **E**, 3′UTR reporter assays of a *miR-195*- and *miR-497*-nonexpressing cell line, Hep G2, 48 hours after cotransfection with pMIR-REPORT luciferase vectors containing 3′-UTR target sites (***upper***) of *CDK6, CCND1, E2F3, CDK4, CCNE1, CCND3, CDC25A*, or *BTRC*, miCENTURY OX miNatural mimicking *miR-195*, *miR-497*, or negative control, and pRL-hTK internal control vector (***lower***). Horizontal open bar, gray boxes, and black bars with arrowheads in upper panel indicate 3′-UTR, possible target sites, and regions examined in the 3′UTR reporter assay, respectively, for each gene.

Since the total numbers of true targets for *miR-195* and *miR-497* in Hep G2 cells are unclear, genes enriched in Ago2IP-deep sequencing with the target sequence were cut off by the most efficient rates with maximum enrichment scores in GSEA. The highest enrichment scores were detected when genes were cut off by top 8% (577 genes) or 36% (2,010 genes) for candidates of *miR-195* or *miR-497* targets, respectively (*p*<0.001 and <0.001, FDR<0.001 and <0.001, and normalized enrichment score = -2.5925288 and -1.9934999 for *miR-195* and *miR-497*, respectively; Fig. 3A and Fig. S4D). The top 20 candidate targets enriched in the Ago2-IP fraction of *miR-195* or *miR-497* combined with target predictions are listed in Table S2.

In order to narrow down promising targets of *miR-195* and *miR-497*, we focused on 190 of 577 and 290 of 2,010 genes remarkably downregulated (>2-fold change) by *miR-195* and *miR-497* transfection in Hep G2 cells (Fig. 3B), respectively, and carried out a pathway analysis. All of those genes were mapped to genetic networks describing functional relationships among gene products based on known interactions as defined by the IPA tool. Consistent with our findings showing that *miR-195* and *miR-497* induced G1 arrest in HCC cells, the IPA tool identified the canonical pathway “Cell Cycle: G1/S Checkpoint Regulation” as a significantly enriched pathway for possible target genes (*p*<0.001 and *p* = 0.00287, respectively; Fig. 3C). Therefore, we focused on candidate genes involved in this pathway, such as *BTRC*, *CCND3*, *CCNE1*, *CDC25A*, *CDK4* and *CDK6* as a target for *miR-195* and *miR-497*, as functionally important direct targets in Hep G2 cells.

### Validation of Possible Direct-targets for *miR-195*/*miR-497* Contributing to Deregulation of Cell Cycle in HCC Cells

**Figure 4:**
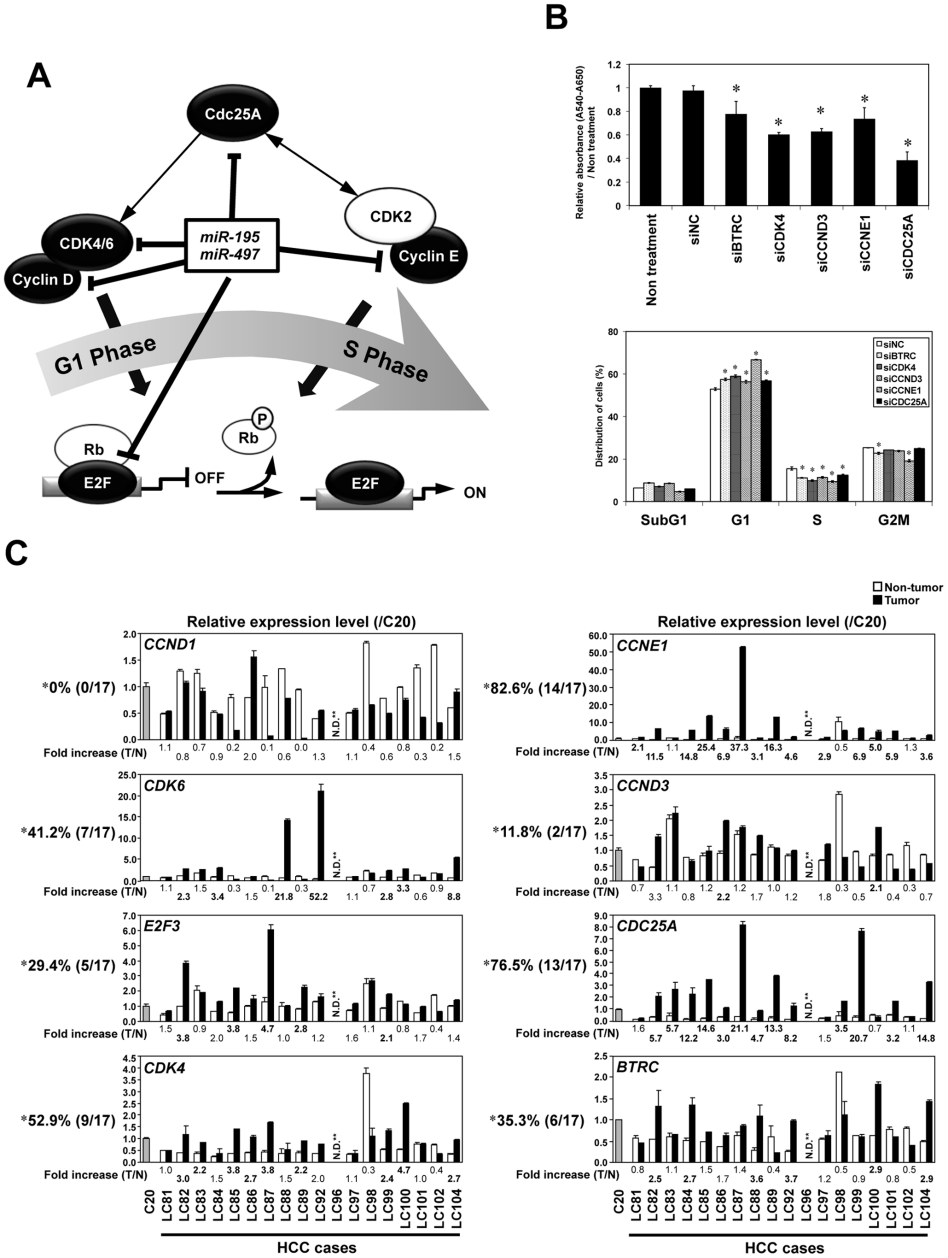
Significance of identified *miR-195* and *miR-497* targets in HCCs. A , Schema of *miR-195* and *miR-497* target genes regulating promotion of G1 to S in the cell cycle. **B,** Results of the knock down of each *miR-195* and *miR-497* target gene by siRNAs. Mean of relative growth ratio of Hep G2 72 hours after transfection of 10 nM of each siRNA (siNC, siBTRC, siCDK4, siCCND3, siCCNE1 or siCDC25A) and non-treated Hep G2 (Non treatment) were assessed by WST-8 assay in a triplicate manner (***upper***). The population in each phase of the cell cycle assessed by FACS were assessed by FACS 48 hours after siRNA transfection (***lower***) in a duplicate manner. Bars, SD. Asterisks (*), *P*<0.05 versus siNC transfected cells in a statistical analysis with the Mann-Whitney *U* test. **C**, Expression status of each target gene in primary HCC tumors (closed boxes) and paired non-tumorous liver tissues (open boxes) as compared with normal liver tissue (C20), which were used in experiments shown in Fig 1D. Asterisk (*), frequencies of primary HCC cases, in which a remarkable up-regulation of possible *miR-195* and/or miR-*497*-target mRNAs was observed in tumor tissues compared with paired non-tumorous liver tissues (>2-fold expression). NA, not available due to lack of available cDNAs for qRT-PCR analyses in the LC96 sample.

Since several cell cycle regulators, such as *CCND1*, *CDK6* and *E2F3*, have been reported as direct targets of *miR-195* in HCC [14], we included them as positive controls in our experiments to validate predicted targets. We first assessed the protein expression of predicted targets 48 hours after transfection with dsRNA mimicking *miR-195* or *miR-497* into Hep G2 and sK-Hep-1 cells (Fig. 3D). A reduction in CDK6 and E2F3 proteins was observed on transfection of each of those miRNAs in both cell lines, whereas no reduction in the CCND1 protein level was observed in Hep G2 cells. Protein levels of CCNE1, BTRC, CDC25A, CCND3 and CDK4 were reduced in both *miR-195* and *miR-497* transfectants compared with their control counterparts. To determine whether *miR-195* and/or *miR-497* directly inhibit the expression of those targets, we performed 3′UTR reporter assays using reporter constructs for each target mRNA containing putative binding sites for these miRNAs (Fig. 3E). Significant reductions in luciferase activity were observed in cells cotransfected with each reporter construct for all 8 genes in *miR-195* or *miR-497* transfectants compared with mock transfectants (Fig. 3E). Taken together, *CCNE1*, *CDC25A*, *CCND3*, *CDK4* and *BTRC* seem to be novel direct targets in addition to three known targets for *miR-195* and *miR-497* in HCC cells (Fig. 4A).

### Significance of Identified *miR-195* and *miR-497* Target Genes in HCCs

We confirm the effect of novel target genes on cell growth by knocking down gene expression using specific small interfering RNAs (siRNA). The knock down efficiency of each gene was determined by Western blotting (Fig. S5). Decreased cell growth with G1 arrest was observed by downregulation of CCNE1, CDC25A, CCND3, CDK4 or BTRC in Hep G2 cells (Fig. 4B), suggesting these genes to be functional targets (Fig. 4A). To assess the consequence of *miR-195/miR-497* and their target genes in HCC cases, we first analyzed the expression of those 8 targets genes in paired primary tissues of HCC used in Fig. 1D by qRT-PCR (Fig. 4B). Among them, only CCNE1, CDC25A and CDK4 showed >2-fold up regulation in over 50% of cases in tumorous compared with non-tumorous tissues. In addition, we also analyzed the correlation between each target gene and *miR-195* or *miR-497* expression using the data set of miRNA expression profiles in 89 primary HCV-related HCCs obtained from the database of the Broad Institute (GSE20596). Surprisingly, only the top-ranked 4 genes, CDK6, CCNE1, CDC25A and CDK4, frequently overexpressed in HCCs showed a slight significant inverse correlation with *miR-195* and *miR-497* expression (p<0.05, correlation coefficient = -0.257, -0.359, -0.315, and -0.355 for *miR-195* and -0.309, -0.299, -0.245, and -0.292 for *miR-497*, respectively; Table S3). In conclusion, CCNE1, CDC25A, and CDK4 together with CDK6 seem to be the most important factors among 8 target genes because of their frequent tumor-specific up-regulation in HCC together with significant inverse correlation with *miR-195* and *miR-497* expression in primary HCCs relevant to cell cycle progression regulated by *miR-195* and *miR-497* in HCCs.

## Discussion

In this study, we used an integrative approach to explore TS-miRNAs contributing to hepatocarcinogenesis by combining function- and expression-based screenings in a genome-wide manner using HCC cell lines, followed by a expression analysis in panels of cell lines and clinical specimens of HCC. Among potentially promising TS-miRNA candidates, we focused on *miR-195* and *miR-497* clustered at 17p13.1. Experimental as well as *in silico* analyses revealed that those miRNAs induced G1 arrest in HCC cells through inhibition of multiple cell cycle regulators as direct targets. We also tested a method for comprehensive identification of miRNA targets by coimmunoprecipitation of mRNAs with miRNA-programmed Ago2-IP-seq, and identified a set of cell cycle regulators including novel targets for *miR-497* and *miR-195*.

Since *miR-195* and *miR-497* are clustered together and their targets largely overlap, these miRNAs seem to be simultaneously transcribed and jointly correlated to the same oncogenic pathways, such as cell cycle regulation in HCC, very efficiently. *MiR-195* and *miR-497* are members of the *miR-15/107* group with the seed sequence AGCAGC, which is important determinant of target recognition [28]. Among members of the *miR-15/107* group, Calin et al. [29] first made the connection between *miR-15a-16* cluster located at 13q14, a region deleted in more than half of B cell chronic lymphocytic leukemias (B-CLL) and several solid tumors, and found that both genes are deleted or downregulated in the majority of CLL. In our screenings using HCC cell lines, *miR-15a* showed TS activity in 4 of 6 cell lines together with significant downregulation in all six cell lines, meeting the requirement for 1st candidate (Fig. 1A), while *miR-16* showed infrequent TS activity and downregulation in HCC cell lines. In addition, *miR-15a* was excluded due to its infrequent tumor-specific downregulation. Among members of the *miR-15/107* group, therefore, *miR-195* and *miR-497* contribute to the pathogenesis of HCC possibly though a tumor-specific dysregulation of expression caused by a tumor-specific histone modification pattern and/or unknown regulatory mechanisms as described in the Supplementary data (Fig. S2–S3).

Our integrated approach for the identification of *miR-195* and *miR-497* targets using Ago2-IP-seq combined with expression analysis revealed that their targets were significantly enriched in cell cycle regulators consistent with their strong activity on cell growth suppression after *miR-195* or *miR-497* overexpression in HCC cell lines. As explained by the concept that the effects of a single miRNA on a specific phenotype in cancer cells are not caused by its strong effect on single target genes but reflect the enrichment of its effects on the large number of target genes involved in a specific signaling pathway [30], [31], only a weak correlation was observed between the expression of each target gene and that of *miR-195* or *miR-497* in the dataset analysis of HCC cases. However, *miR-195* and *miR-497*, can coordinately regulate many aberant components involved in the pathway of cell cycle regulation through their multi-gene-targeting capability.

Meanwhile, among the target genes for *miR-195* and *miR-497* identified in the present study, top four target genes, *CCNE1*, *CDC25A*, *CDK4* and *CDK6*, frequently upregulated in a tumor-specific manner only showed slight inverse correlation with these miRNAs in HCC tumors. These results show the novel possibility for miRNAs that, these miRNAs could preferentially target genes aberrantly expressed in HCCs when restored.

Most cancer cells show excessive proliferation with promoted expression of cell cycle regulators. Since master regulators of the cell cycle are also indispensable for normal cells, current anti-cancer therapeutic strategies have shifted to the search for single dominant oncogenes or addictive molecules showing oncogene addiction [3]. However, several issues, such as the emergence of escape mechanisms, drug resistance and tumor-individuality arising among patients without optimal ways for personalized cancer therapy, remain to be solved for successful translation of this oncogene addiction model into the effective targeted therapies. Therefore, *miR-195* and *miR-497*, may be useful for selective therapeutics, because the introduction of those miRNAs, which are essential and expressed in normal cells at basal levels, may selectively modulate aberrant tumor cell growth without gain of resistance by targeting a set of genes contributing to cell cycle progression which is commonly appeared to be enhanced in tumors. A better understanding on the functions of miRNAs in HCC would undoubtedly open new avenues for research in tumor biology.

## Materials and Methods

### Cell Lines and Primary Tumor Samples

A total of 19 HCC cell lines were maintained in appropriate media as described elsewhere [32]. To examine the restoration of mRNA expression in genes of interest, the cell lines were cultured with or without 5 µM 5-aza-2′-deoxycytidine (5-aza-dCyd) for 5 days and/or 300 nM of TSA for the last 24 hours.

A total of 18 frozen primary tumor samples and corresponding non-tumorous tissue samples were obtained from HCC patients, and two frozen normal liver tissues samples (C20, C40) were obtained from patients with hepatectomy due to metastatic liver tumor treated at Tokyo Medical and Dental University with written consent from each patient and approval by the local ethics committees of Medical Research Institute and Faculty of Medicine, TMDU. The TNM classification of the Union for International Cancer Control (UICC) was used.

### Function-based miRNA Screening

HCC cells were seeded in 24-well plates a day before transfection. Each synthetic miRNA (5 nM) in Pre-miR™ miRNA Precursor Library-Human V3 (Ambion, Austin, TX) or the control non-specific miRNA (Ambion) was transfected into cells using Lipofectamine™ RNAiMAX (Invitrogen, Carlsbad, CA) according to the manufacturer’s instructions. The numbers of viable cells were assessed 4 or 5 days after transfection by the colorimetric water-soluble tetrazolium salt (WST) assay. Results were normalized to the number of cells transfected with non-specific miRNA. Each assay was performed in duplicate.

### Microarray Analyses

Extracted total RNA was subjected to analysis using G4471A 15K Human miRNA Microarrays Ver. 3 and G4112F 44K Whole Human Genome Microarray for miRNA and mRNA expression, respectively, with standard image acquisition according to the manufacturer’s instructions (Agilent Technologies, Santa Clara, CA). Raw microarray data were normalized and analyzed using GeneSpring GX software version 11.5.1 (Agilent Technologies). All samples were analyzed twice.

### Quantitative Reverse Transcription-PCR (qRT-PCR)

Real-time qRT-PCR was performed using an ABI Prism 7500 Fast Real-time PCR System (Applied Biosystems, Foster City, CA), KAPA PROBE FAST Universal 2×qPCR master mix (Kapa Biosystems, Woburn, MA), TaqMan® Reverse Transcription Kit (Applied Biosystems), and TaqMan® MicroRNA Assays (Applied Biosystems) according to the manufacturer’s instructions [33]. All reactions were run in duplicate.

### Transfection with Synthetic miRNAs or siRNAs and Proliferation Assay

Each of miCENTURY OX miNatural mimicking *miR-195*, *miR-497*, or “microRNA control, Luciferase (GL3)” (5 nM; COSMO BIO, Tokyo, Japan) was efficiently transfected into cells as described elsewhere [33]. Each of gene-specific siRNA (Hs_CCNE1_0049, Hs_CDC25A_7714, Hs_CCND3_0184, Hs_CDK4_2490, and Hs_BTRC_9438; 10 nM; Sigma, St Louis, MO) or negative control siRNA (Mission_Negative control SIC-001, Sigma) was also efficiently transfected into cells. The numbers of viable cells 24–96 hours after transfection were assessed by WST assay. The cell cycle was evaluated 48 hours after transfection by fluorescence-activated cell sorting (FACS) as described [33].

### Ago2-IP and RNA Deep Sequencing

Ago2-IP was performed using 1×10^7^ cells 24 hours after transfection of miCENTURY OX miNatural mimicking *miR-195* or *miR-497*, or non-transfected cells, using Ago2 antibody and a microRNA Isolation Kit, human Ago2 (Wako Pure Chemical, Osaka, Japan) according to the manufacturer’s instructions. Samples for RNA-sequencing were prepared using 10 ng of Ago2-IP RNA and 3 µg of total RNA with a TrueSeq mRNA Sample Preparation Kit (illumina, San Diego, CA) with or without poly-A selection. Each of the libraries were analyzed by Genome Analyzer IIx (illumina) according to the manufacturer’s instructions. The 36-base single-read sequences were mapped to human genomic sequences (hg19) using the sequence alignment program ELANDv2. For the expression analysis, sequence reads were converted to RPKM (Reads Per Kilobase per Million mapped reads) values by using CASAVA 1.7 software (illumina).

### miRNA Target Predictions

Predicted targets of *miR-195* and *miR-497* and their sites were analyzed by searching for a possible targeting sequence (5′-ACGACGA-3′) in the 3′UTR of mRNA using the Ensembl Genome Browser (http://asia.ensembl.org/Homo_sapiens/) and Ensembl Perl API as well as by searching databases, such as MicroCosm Targets Version 5 (http://www.ebi.ac.uk/enright-srv/microcosm/htdocs/targets/v5/), microRNA.org (http://www.microrna.org/), and TargetScan (http://www.targetscan.org/).

### Bioinformatics Analysis for Identification of miRNA Targets

Cut-off values of Ago2-IP-seq were calculated for genes with miRNA target predictions using Gene Set Enrichment Analysis (GSEA; www.broad.mit.edu/gsea). Candidate target genes were selected as an intersect of Ago2-IP-seq final candidates and downregulated genes by miRNA transfection, followed by pathway analyses using Ingenuity Pathway Analysis (IPA; Ingenuity Systems, Inc., Redwood City, CA). Detailed information for each analysis is provided in Methods S1.

### Western Blotting and 3′UTR Reporter Assay

Western blotting and 3′UTR reporter assays were performed as described elsewhere [33]. In the 3′UTR reporter assay, “microRNA control, Non-target RNA” (COSMO BIO) was used as a negative control. PCR primers designed to amplify regions of interests, and antibodies used for Western blotting are provided in Table S4 and Methods S1.

### Data Deposition

The microarray and sequencing data from this publication have been submitted to the GEO database (http://www.ncbi.nlm.nih.gov/geo/) and assigned the identifier “GSE41081”.

## Supporting Information

Figure S1 Expression levels of seven candidate miRNAs in HCC cell lines (**A**) and in primary HCC tumors (closed boxes, **B**) and paired non-tumorous liver tissues (open boxes, **B**) as compared with normal liver tissue (C20). Asterisks (*), frequencies of HCC cell lines in which a remarkable downregulation of the expression of candidate miRNAs was observed as compared with C20 (<0.5-fold expression). Numbers under the boxes in the right panel indicate the fold increase of the expression in tumors (T) compared with paired non-tumorous liver tissue (N). Double asterisks (**), frequencies of primary HCC cases in which a remarkable down-regulation of candidate miRNA expression in tumors was observed compared with paired non-tumorous liver tissue (<0.5-fold expression).
(PDF)

Figure S2 Analysis of expression and genomic aberrations of *miR-497* and *miR-195.* **A**, Scatter plot showing *miR-195* (x-axis) and *miR-497* (y-axis) expression status in the HCV-related HCC dataset (tumor only, ***left***) obtained from the database of Broad institute (GSE20596, http://www.broadinstitute.org/) and in our dataset used in Fig. S1B (tumors and non-tumorous tissues are filled and open plots, respectively; ***right***). Pearson’s product moment correlation coefficient (*R*) was used to assess this relationship. **B**, Genomic copy-number status around *miR-195* and *miR-497* genes determined by q-gPCR. The copy-number of *COL7A1* at 3p21.31 was used for normalization, and all results were shown as copy-number ratios relative to those of CONT1. CONT1-6 (DNA samples from normal lymphoblastoid cells) and C20 (DNA sample from normal liver) were used as normal controls. We used the data on genomic copy-numbers in regions around *miR-195*, *miR-497* and *COL7A1* in some HCC cell lines (HLE, HuH-6, Huh7, PLC/PRF/5) determined by single nucleotide polymorphism (SNP) arrays available online in SNP Array Based LOH and Copy Number Analysis of the Sanger Center Genome Project (http://www.sanger.ac.uk/genetics/CGP/) to confirm our results. *LOH was detected at *miR-195* and *miR-497* loci, **copy number changes were observed neither at *miR-195* and *miR-497* nor *COL7A1* loci, ***LOH was observed both at *miR-195* and *miR-497* and *COL7A1* alleles in the Sanger Center Genome Project, which was consistent with our result. C, Expression levels of *pri-miRNA-195* and *pri-miRNA-497* in HCC cell lines as compared with normal liver tissue (C20).
(PDF)

Figure S3 Assessment of possible mechanisms causing downregulation of *miR-195* and *miR-497* in HCC cell lines. **A**, Schematic map of *miR-497-195* loci at 17p13.1 (black thick bar). White arrows indicate positions of *pre-miR-497* and *pre-miR-195* coding region. SwitchGear Genomics Transcription Start Sites (CHR17_M0118_R1) was marked at +1 (thin arrow). Hatched bar indicates the site of an ENCODE Transcription Factor ChIP-seq (TAF1) binding site. The regions used for promoter assay, bisulfite sequencing and ChIP-PCR are indicated white or dotted boxes, closed arrows, and gray bars, respectively. Dotted boxes indicated regions showing promoter activity in the promoter assay. **B**, Promoter assay of regions around the predicted *pri-miR-497-195* transcription start site. pGL3 basic empty vectors (mock) or constructs containing sequences of F1–4 (see Fig. S3A) were transfected into HCC cell lines without expression of *miR-497* and *miR-195* (Hep G2 and sK-Hep-1, ***left***) or with expression of *miR-195* (HuH-6, ***right***). The Luciferase activity of each construct relative to the values of pGL3-basic empty vector is shown with the mean ± SD (bars) in triplicate experiments. **C**, Effect of treatment with 5-aza-2′-deoxycytidine (5-aza-dCyd) (5 µmol/L) for 5 days and/or trichostatin A (TSA) (300 nmol/L) for the last 24 hours on expression levels of *miR-195* (***left***) and *miR-497* (***right***) in HCC cell lines determined by qRT-PCR. Results are shown with means ± SDs (bars) relative to the values for no treatment in duplicate experiments for each cell line. **D**, Representative results of bisulfite-sequencing in regions 1–5 upstream of *miR-497* and *miR-195* in *miR-497/195*-expressing normal liver (C20, ++), *miR-195*-expressing HCC cell line (+), and non-expressing HCC cell lines (−). Each circle indicates the position of the CpG site in the region. Methylated- and unmethylated-CpG sites are shown as closed and open circles, respectively. **E**, Representative results of ChIP assays showing H3K27me3 status (upper) and H3K4me3 status(lower) indicating inactive and active transcription status, respectively, within regions R1 and R2 (see Fig. S3A) in non-expressing cells(−) and *miR-195*-expressing cell (+). Results are shown with means ± SDs (bars) relative to the values for ChIP with anti-H3 antibody in duplicate experiments for each cell line.
(PDF)

Figure S4 Validation and determination of the cut-off value for Ago2-IP experiments. **A**, Scatter plot of the miRNA expression profile in the Ago2-IP fraction for *miR-195* (***left***, y-axis) and *miR-497* (***right***, y-axis) overexpression compared with non-transfected (NT, x-axis) samples determined by miRNA expression array. Closed and open blocks indicate expression of *miR-195* and *miR-497*, respectively. **B**, GSEA profile of the Running Enrichment Score (ES) of genes shown in the top 10% of fold enrichment scores in Ago2-IP experiments for *miR-195* (***left***) or *miR-497* (***right***) along the rank of transcripts differentially expressed in *miR-195-* (***left***) or *miR-497-* (***right***) overexpression compared with control counterparts. **C**, Results of the same analysis as in Figure S3B using different gene sets, which were further selected by the presence of *miR-195* or *miR-497* predicted target sites (***left***, *miR-195*; ***right***, *miR-497*). **D**, Normalized enrichment score calculated by GSEA for all cut-off rates (%). Dotted lines indicate the cut off rates, which show maximum negative enrichment scores (8 and 36% for *miR-195* and *miR-497* experiments, respectively).
(PDF)

Figure S5 Representative results of Western blotting of CDK4, CCNE1, CCND3, CDC25A, and BTRC 48 hours after transfection with each specific siRNA or negative control (siNC).
(PDF)

Table S1 Primers for 3′UTR reporter assay.
(ZIP)

Table S2 GO analysis of genes, whose expression levels were changed (>2-fold change) by *miR-195* or *miR-497* overexpression compared to control *Luc*-overexpression in Hep G2 (S2-1 or S2-2) or sK-Hep-1 (S2-3 or S2-4) cells 48 hours after transfection.
(PDF)

Table S3 Top 20 genes ranked by the fold enrichment score in Ago2-IP-seq with miRNA target predictions.
(PDF)

Table S4 Significance of identified candidate targets for *miR-195* and *miR-497* through Ago2-IP-seq, expression array experiments, and statistical analyses.
(PDF)

Table S5 Primer sets for q- gPCR, promoter assay, bisulfite sequencing, and ChIP-PCR.
(PDF)

Methods S1 (DOC)

Dataset S1 List of candidates for growth-suppressive miRNAs determined by function-based screening.
(XLSX)

Dataset S2 List of downregulated miRNAs in 6 HCC cell lines compared with C20 and C40 determined by miRNA expression array.
(XLSX)
