# Supplementary figures and images for: The Tumor-Suppressive miR-497-195 Cluster Targets Multiple Cell-Cycle Regulators in Hepatocellular Carcinoma
Source: PLoS One. 2013 Mar 27;8(3):e60155. doi: 10.1371/journal.pone.0060155 (PMC3609788; doi:10.1371/journal.pone.0060155)

Supplementary Fig. S1

A

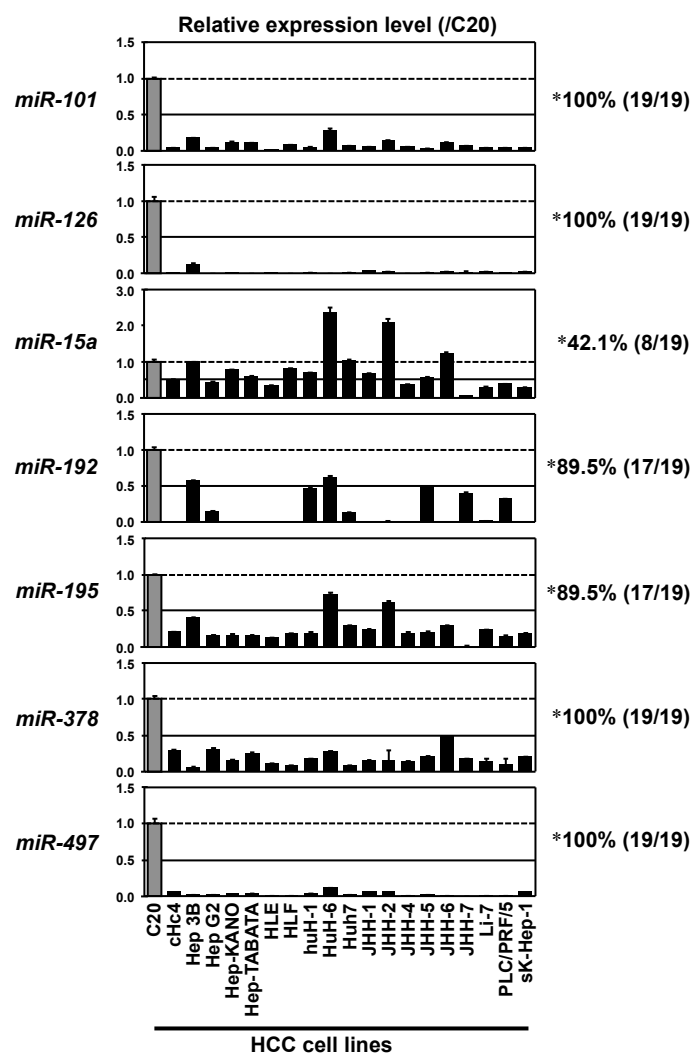

Supplementary Fig. S1 CONTINUED

B

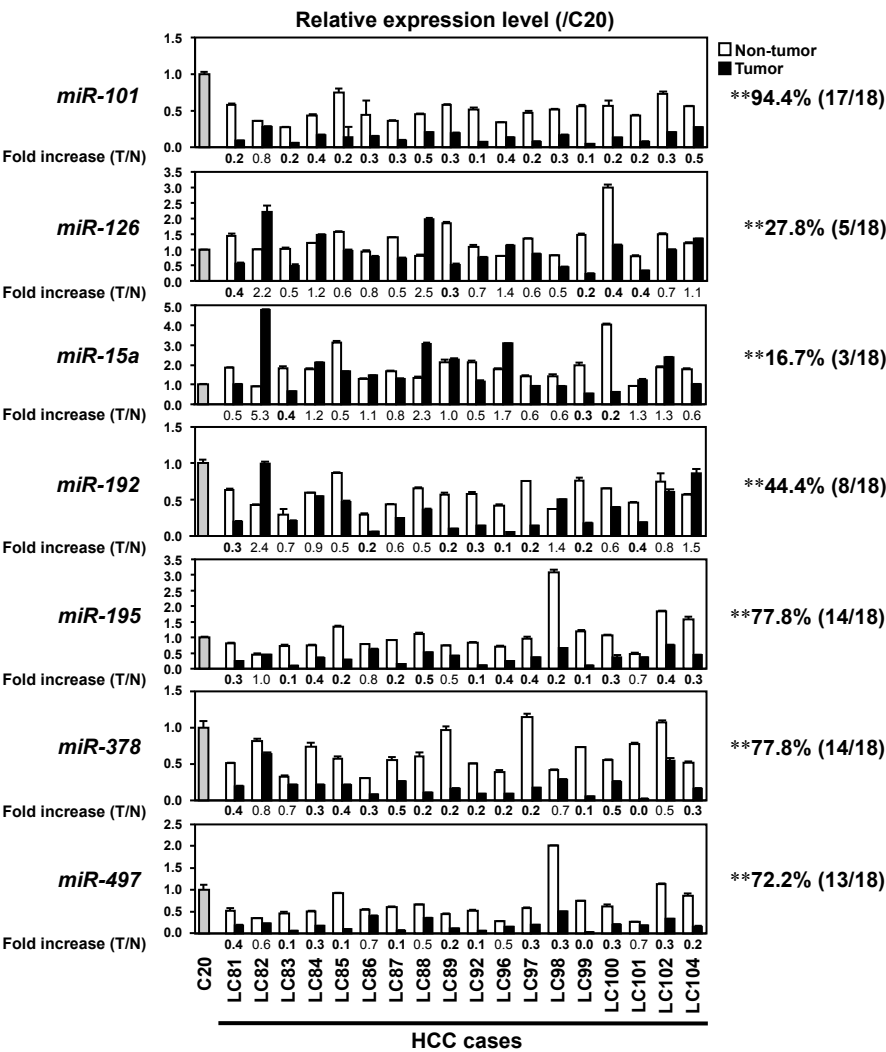

Supplement: Figure S1 — Expression levels of seven candidate miRNAs in HCC cell lines (A) and in primary HCC tumors (closed boxes, B) and paired non-tumorous liver tissues (open boxes, B) as compared with normal liver tissue (C20). Asterisks (*), frequencies of HCC cell lines in which a remarkable downregulation of the expression of candidate miRNAs was observed as compared with C20 (<0.5-fold expression). Numbers under the boxes in the right panel indicate the fold increase of the expression in tumors (T) compared with paired non-tumorous liver tissue (N). Double asterisks (**), frequencies of primary HCC cases in which a remarkable down-regulation of candidate miRNA expression in tumors was observed compared with paired non-tumorous liver tissue (<0.5-fold expression). (PDF) [file pone.0060155.s001.pdf]

Supplementary Fig. S2

A

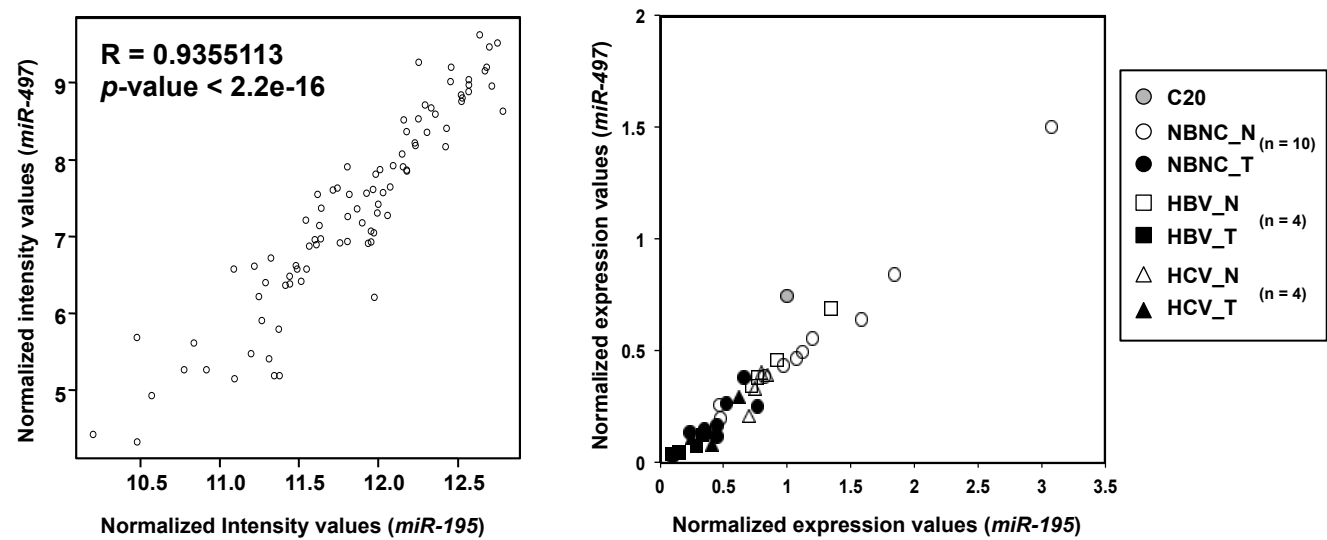

B

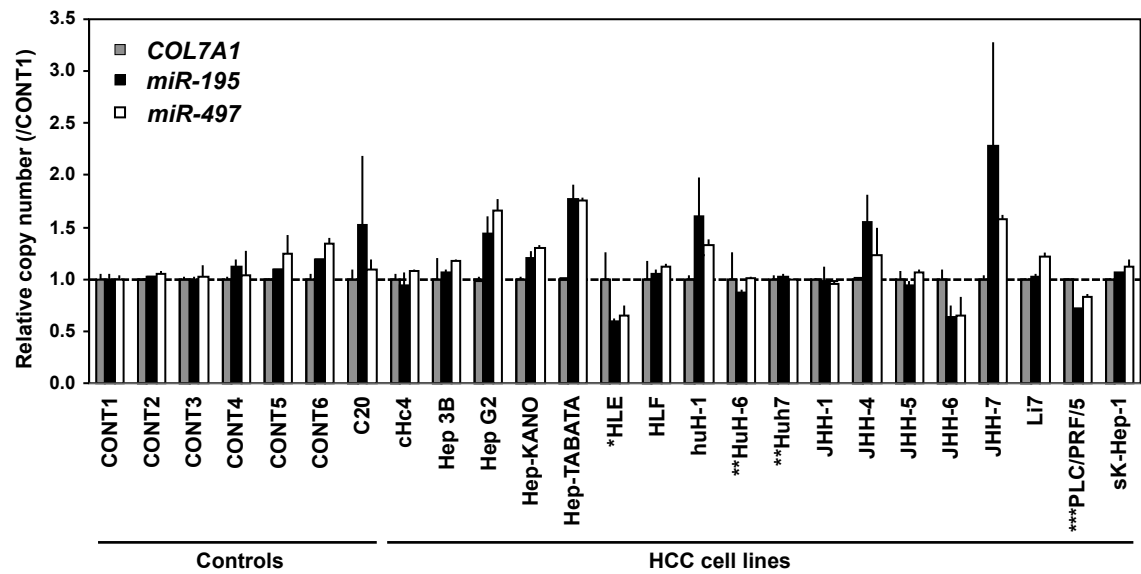

C

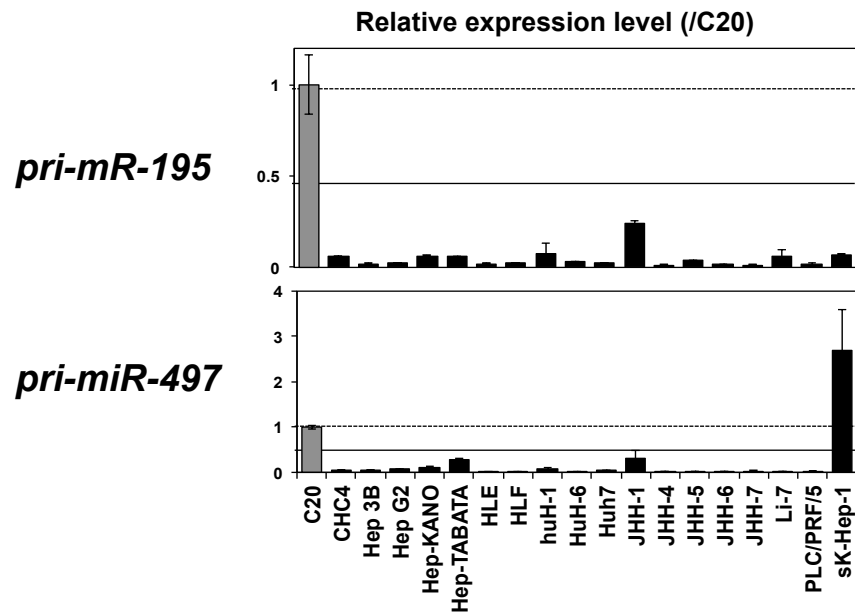

Supplement: Figure S2 — Analysis of expression and genomic aberrations of miR-497 and miR-195. A, Scatter plot showing miR-195 (x-axis) and miR-497 (y-axis) expression status in the HCV-related HCC dataset (tumor only, left) obtained from the database of Broad institute (GSE20596, http://www.broadinstitute.org/) and in our dataset used in Fig. S1B (tumors and non-tumorous tissues are filled and open plots, respectively; right). Pearson’s product moment correlation coefficient (R) was used to assess this relationship. B, Genomic copy-number status around miR-195 and miR-497 genes determined by q-gPCR. The copy-number of COL7A1 at 3p21.31 was used for normalization, and all results were shown as copy-number ratios relative to those of CONT1. CONT1-6 (DNA samples from normal lymphoblastoid cells) and C20 (DNA sample from normal liver) were used as normal controls. We used the data on genomic copy-numbers in regions around miR-195, miR-497 and COL7A1 in some HCC cell lines (HLE, HuH-6, Huh7, PLC/PRF/5) determined by single nucleotide polymorphism (SNP) arrays available online in SNP Array Based LOH and Copy Number Analysis of the Sanger Center Genome Project (http://www.sanger.ac.uk/genetics/CGP/) to confirm our results. *LOH was detected at miR-195 and miR-497 loci, **copy number changes were observed neither at miR-195 and miR-497 nor COL7A1 loci, ***LOH was observed both at miR-195 and miR-497 and COL7A1 alleles in the Sanger Center Genome Project, which was consistent with our result. C, Expression levels of pri-miRNA-195 and pri-miRNA-497 in HCC cell lines as compared with normal liver tissue (C20). (PDF) [file pone.0060155.s002.pdf]

Supplementary Fig. S3

A

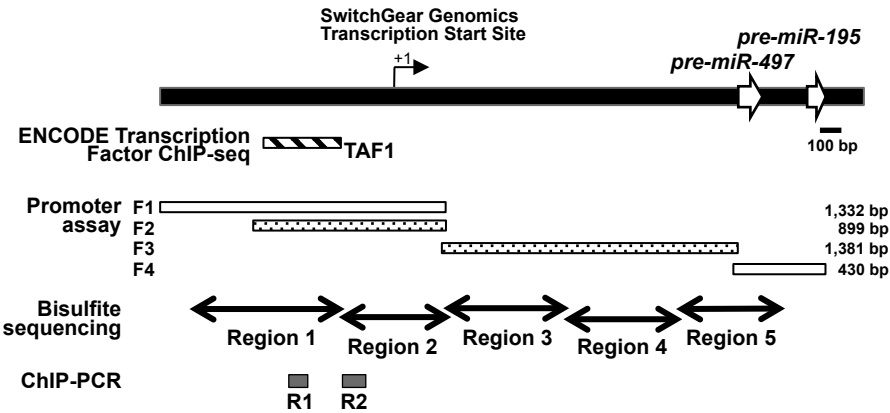

B

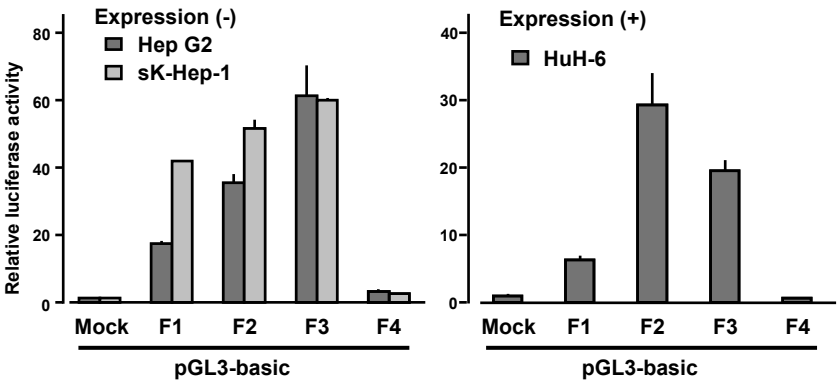

C

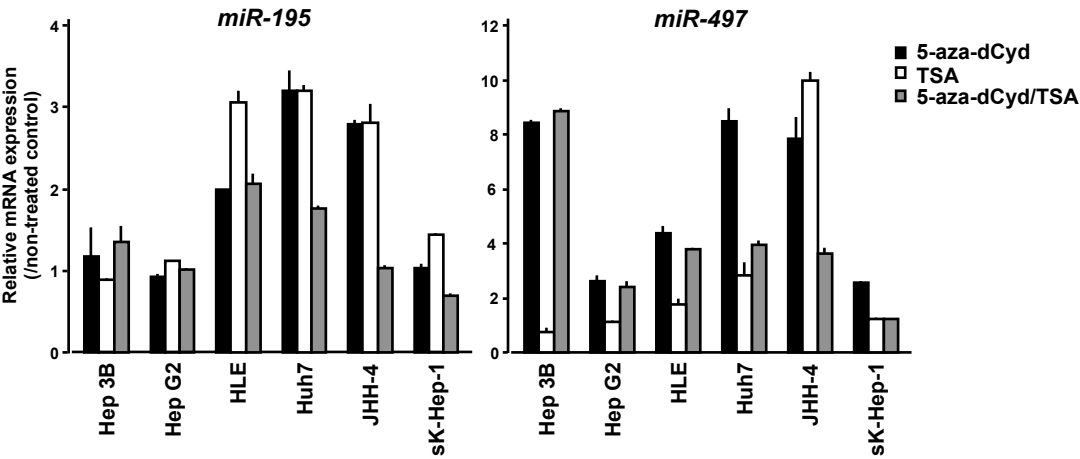

Supplementary Fig. S3 CONTINUED

D

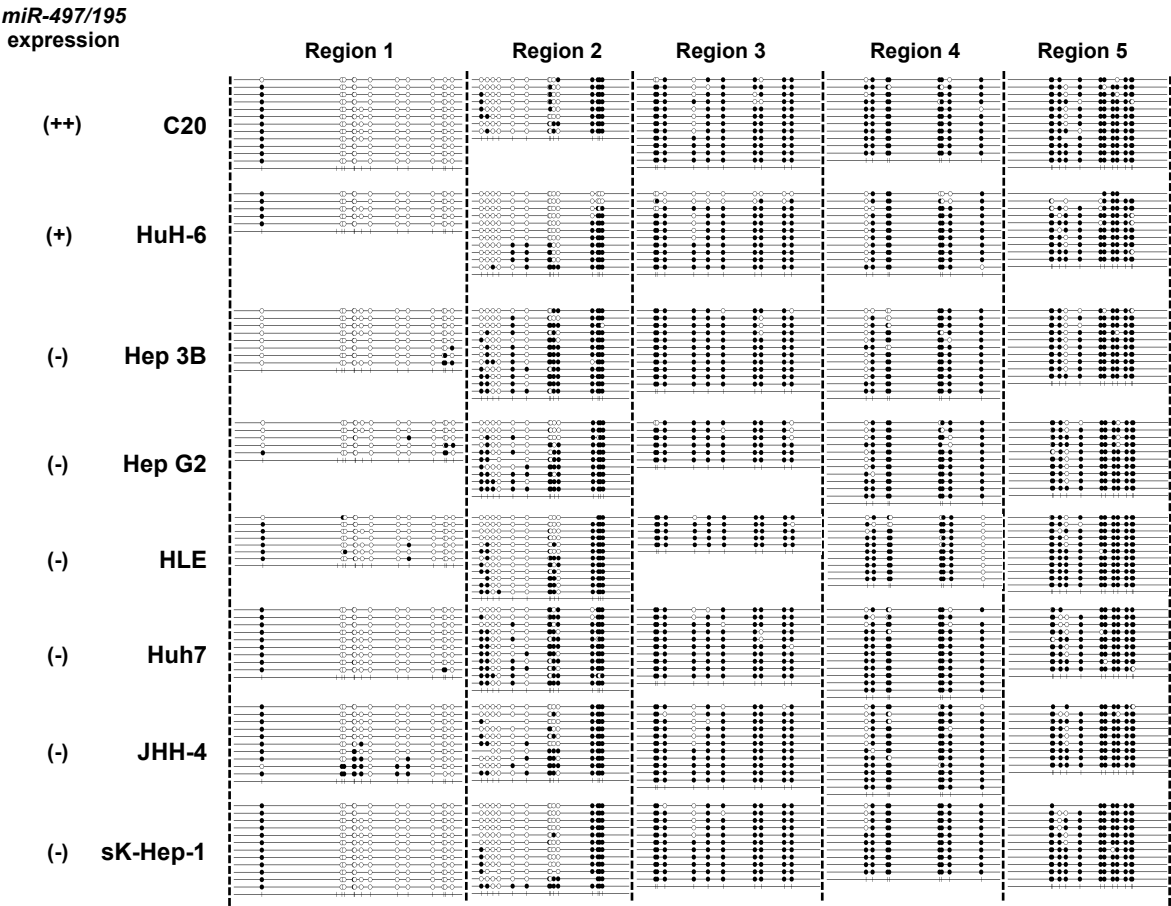

E

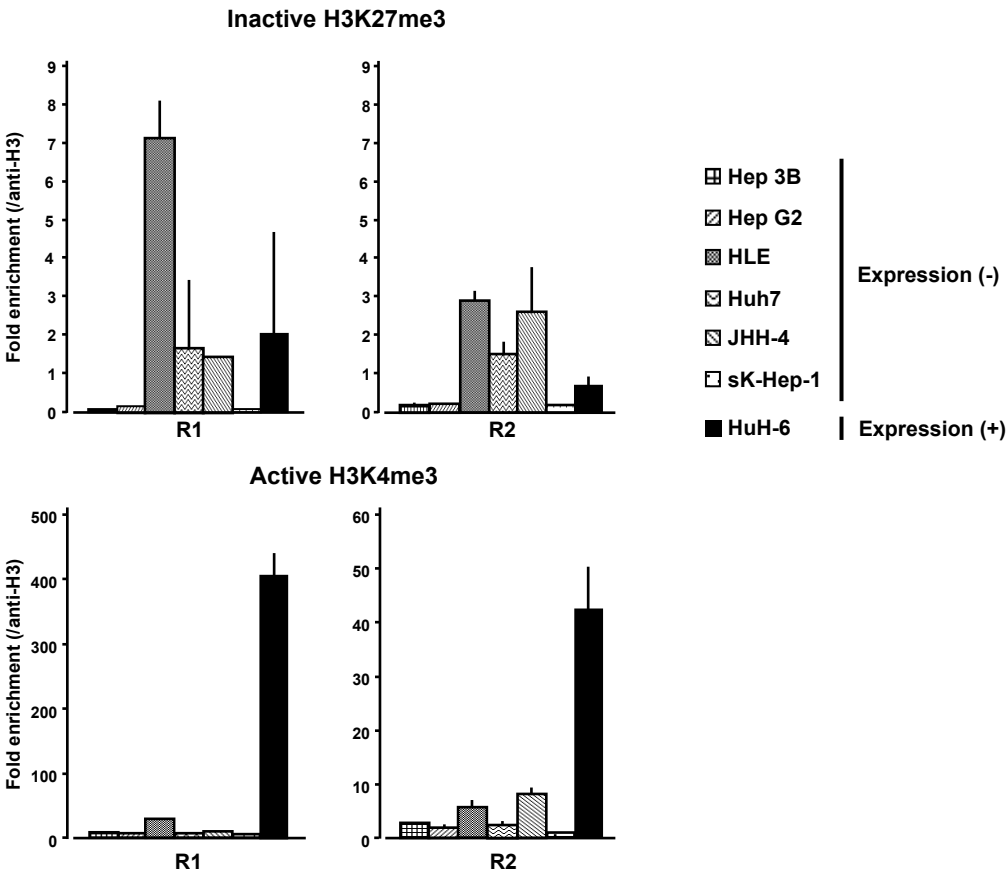

Supplement: Figure S3 — Assessment of possible mechanisms causing downregulation of miR-195 and miR-497 in HCC cell lines. A, Schematic map of miR-497-195 loci at 17p13.1 (black thick bar). White arrows indicate positions of pre-miR-497 and pre-miR-195 coding region. SwitchGear Genomics Transcription Start Sites (CHR17_M0118_R1) was marked at +1 (thin arrow). Hatched bar indicates the site of an ENCODE Transcription Factor ChIP-seq (TAF1) binding site. The regions used for promoter assay, bisulfite sequencing and ChIP-PCR are indicated white or dotted boxes, closed arrows, and gray bars, respectively. Dotted boxes indicated regions showing promoter activity in the promoter assay. B, Promoter assay of regions around the predicted pri-miR-497-195 transcription start site. pGL3 basic empty vectors (mock) or constructs containing sequences of F1–4 (see Fig. S3A) were transfected into HCC cell lines without expression of miR-497 and miR-195 (Hep G2 and sK-Hep-1, left) or with expression of miR-195 (HuH-6, right). The Luciferase activity of each construct relative to the values of pGL3-basic empty vector is shown with the mean ± SD (bars) in triplicate experiments. C, Effect of treatment with 5-aza-2′-deoxycytidine (5-aza-dCyd) (5 µmol/L) for 5 days and/or trichostatin A (TSA) (300 nmol/L) for the last 24 hours on expression levels of miR-195 (left) and miR-497 (right) in HCC cell lines determined by qRT-PCR. Results are shown with means ± SDs (bars) relative to the values for no treatment in duplicate experiments for each cell line. D, Representative results of bisulfite-sequencing in regions 1–5 upstream of miR-497 and miR-195 in miR-497/195-expressing normal liver (C20, ++), miR-195-expressing HCC cell line (+), and non-expressing HCC cell lines (−). Each circle indicates the position of the CpG site in the region. Methylated- and unmethylated-CpG sites are shown as closed and open circles, respectively. E, Representative results of ChIP assays showing H3K27me3 status (upper) and H3K4me3 stat [file pone.0060155.s003.pdf]

Supplementary Fig. S4

A

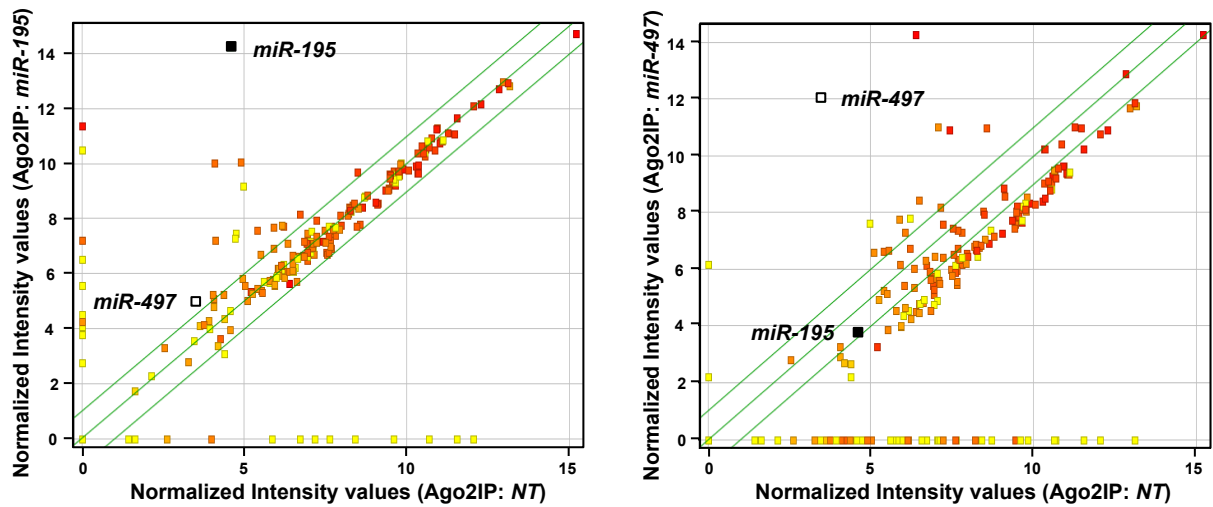

B

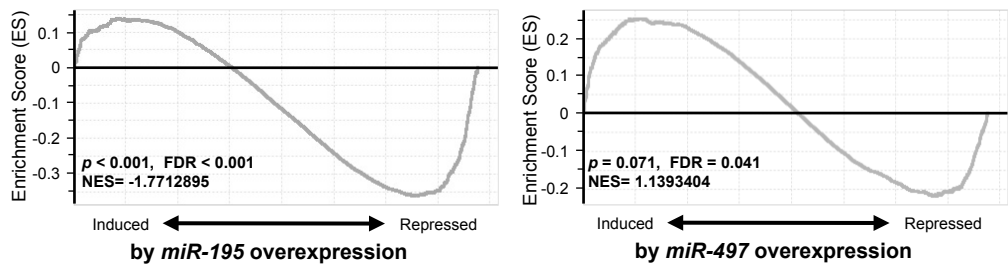

C

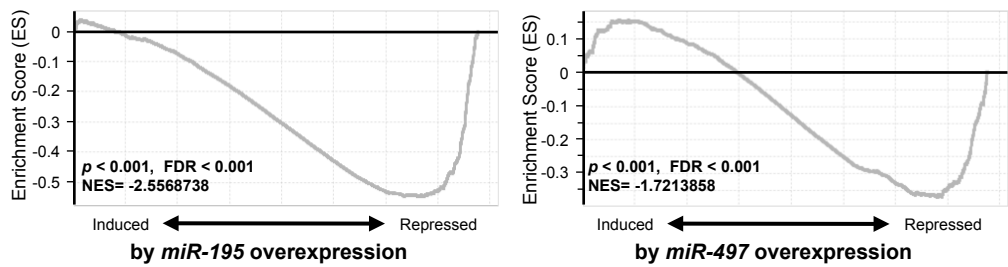

D

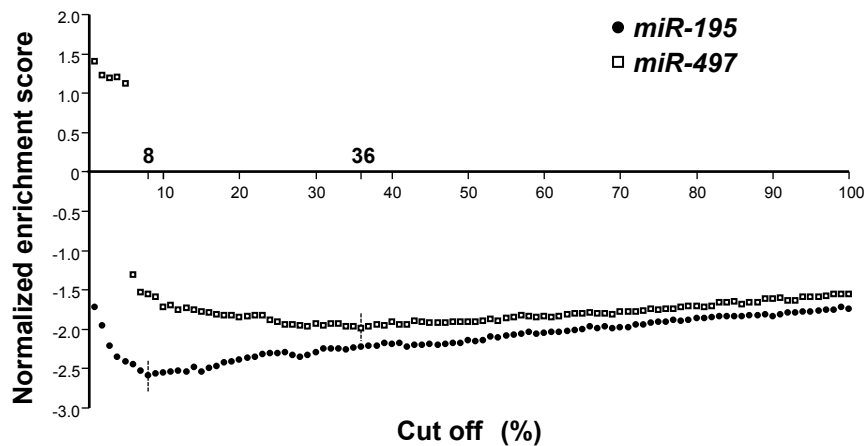

Supplement: Figure S4 — Validation and determination of the cut-off value for Ago2-IP experiments. A, Scatter plot of the miRNA expression profile in the Ago2-IP fraction for miR-195 (left, y-axis) and miR-497 (right, y-axis) overexpression compared with non-transfected (NT, x-axis) samples determined by miRNA expression array. Closed and open blocks indicate expression of miR-195 and miR-497, respectively. B, GSEA profile of the Running Enrichment Score (ES) of genes shown in the top 10% of fold enrichment scores in Ago2-IP experiments for miR-195 (left) or miR-497 (right) along the rank of transcripts differentially expressed in miR-195- (left) or miR-497- (right) overexpression compared with control counterparts. C, Results of the same analysis as in Figure S3B using different gene sets, which were further selected by the presence of miR-195 or miR-497 predicted target sites (left, miR-195; right, miR-497). D, Normalized enrichment score calculated by GSEA for all cut-off rates (%). Dotted lines indicate the cut off rates, which show maximum negative enrichment scores (8 and 36% for miR-195 and miR-497 experiments, respectively). (PDF) [file pone.0060155.s004.pdf]

Supplementary Fig. S5

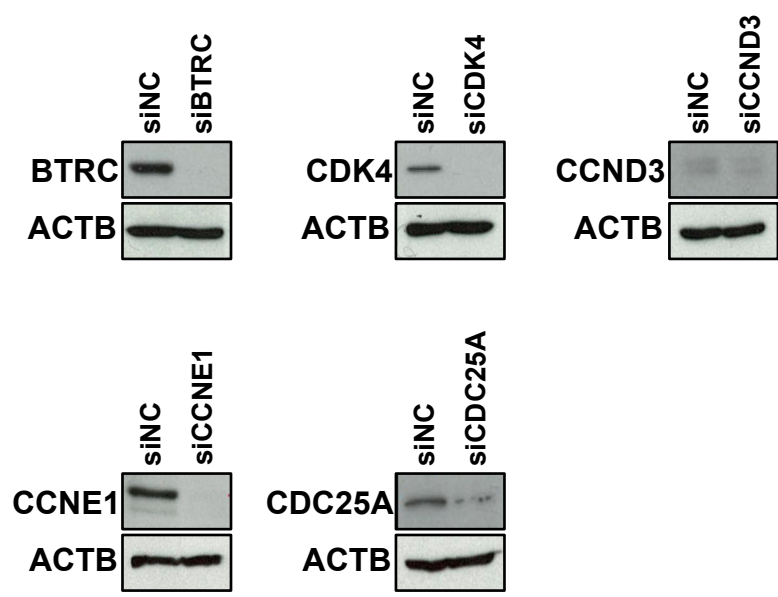

Supplement: Figure S5 — Representative results of Western blotting of CDK4, CCNE1, CCND3, CDC25A, and BTRC 48 hours after transfection with each specific siRNA or negative control (siNC). (PDF) [file pone.0060155.s005.pdf]
