# Supplementary material for: The Tumor-Suppressive miR-497-195 Cluster Targets Multiple Cell-Cycle Regulators in Hepatocellular Carcinoma
Source: PLoS One. 2013 Mar 27;8(3):e60155. doi: 10.1371/journal.pone.0060155 (PMC3609788; doi:10.1371/journal.pone.0060155)
Supplement: Table S1 — Primers for 3′UTR reporter assay. (ZIP) [file pone.0060155.s006.zip › Sup.tableS1-1.pdf]

Supplementary Table 1-1 Gene ontology analysis of genes, whose expression levels were changed by *miR-195* overexpression in Hep G2 (fold change > 2.0 in *miR-195*-overexpressed cells compared with *Luc*-overexpressed cells, 48 hours)

GO accession	GO term	*p*-value[c]	Corrected *p*-value[c]	Genes in selection[d]		Genes in total[e]	
				count[a]	%[b]	count[a]	%[b]
GO:0007049	**cell cycle**	3.48E-21	2.41E-16	202	12.1467	729	4.4942
GO:0022403	**cell cycle phase**	2.39E-20	8.27E-16	95	5.7126	397	2.4474
GO:0022402	**cell cycle process**	1.05E-19	2.42E-15	96	5.7727	541	3.3352
GO:0000279	**M phase**	9.46E-18	1.64E-13	94	5.6524	321	1.9789
GO:0006260	**DNA replication**	2.36E-16	3.27E-12	79	4.7505	186	1.1467
GO:0007067	**mitosis**	4.86E-15	4.81E-11	91	5.4720	215	1.3254
GO:0000278	**mitotic cell cycle**	4.21E-15	4.81E-11	93	5.5923	355	2.1885
GO:0000087	**M phase of mitotic cell cycle**	7.76E-15	6.71E-11	93	5.5923	219	1.3501
GO:0000793	**condensed chromosome**	1.89E-14	1.45E-10	35	2.1046	124	0.7644
GO:0051301	**cell division**	1.21E-13	8.35E-10	106	6.3740	279	1.7200
GO:0005694	**chromosome**	6.02E-13	3.79E-09	105	6.3139	433	2.6694
GO:0000775	**chromosome, centromeric region**	1.27E-12	7.32E-09	51	3.0667	119	0.7336
GO:0000779	**condensed chromosome, centromeric region**	3.27E-12	1.74E-08	28	1.6837	64	0.3946
GO:0006259	**DNA metabolic process**	1.20E-11	5.92E-08	125	7.5165	469	2.8913
GO:0044427	**chromosomal part**	3.41E-11	1.57E-07	56	3.3674	358	2.2070
GO:0000777	**condensed chromosome kinetochore**	1.04E-10	4.21E-07	27	1.6236	57	0.3514
GO:0005819	**spindle**	9.86E-11	4.21E-07	51	3.0667	142	0.8754
GO:0015630	**microtubule cytoskeleton**	1.91E-10	7.34E-07	118	7.0956	516	3.1811
GO:0043232	**intracellular non-membrane-bounded organelle**	3.36E-10	1.16E-06	324	19.4829	2387	14.7155
GO:0043228	**non-membrane-bounded organelle**	3.36E-10	1.16E-06	324	19.4829	2387	14.7155
GO:0007059	**chromosome segregation**	1.01E-09	3.31E-06	27	1.6236	80	0.4932
GO:0005622	**intracellular**	3.42E-09	1.08E-05	1613	96.9934	10397	64.0959
GO:0000776<br>GO:0005699	**kinetochore**	4.34E-09	1.30E-05	32	1.9242	75	0.4624
GO:0044422	**organelle part**	6.26E-09	1.80E-05	207	12.4474	3953	24.3696
GO:0044446	**intracellular organelle part**	1.44E-08	3.98E-05	206	12.3873	3939	24.2833
GO:0044424	**intracellular part**	2.18E-08	5.80E-05	1486	89.3566	10071	62.0862
GO:0044430	**cytoskeletal part**	6.62E-08	1.70E-04	114	6.8551	847	5.2216
GO:0005874	**microtubule**	7.05E-08	1.74E-04	93	5.5923	254	1.5659
GO:0007017	**microtubule-based process**	1.21E-07	2.90E-04	14	0.8419	250	1.5412
GO:0005856	**cytoskeleton**	1.67E-07	3.85E-04	227	13.6500	1260	7.7677
GO:0005737	**cytoplasm**	6.86E-07	0.00153	1091	65.6043	7017	43.2587
GO:0006950	**response to stress**	1.32E-06	0.002852	123	7.3963	1474	9.0870
GO:0000070<br>GO:0016359	**mitotic sister chromatid segregation**	1.50E-06	0.003154	7	0.4209	35	0.2158
GO:0043226	**organelle**	2.88E-06	0.005859	986	59.2904	8491	52.3457
GO:0000819	**sister chromatid segregation**	2.98E-06	0.005884	7	0.4209	36	0.2219
GO:0043229	**intracellular organelle**	3.33E-06	0.006403	986	59.2904	8478	52.2656
GO:0005657	**replication fork**	5.16E-06	0.009643	5	0.3007	32	0.1973
GO:0044428	**nuclear part**	5.56E-06	0.010132	50	3.0066	1728	10.6529
GO:0006996	**organelle organization and biogenesis**	6.08E-06	0.010781	43	2.5857	1343	8.2794
GO:0007010	**cytoskeleton organization and biogenesis**	6.33E-06	0.010953	27	1.6236	532	3.2797
GO:0046983	**protein dimerization activity**	6.56E-06	0.011074	29	1.7438	504	3.1071
GO:0051726<br>GO:0000074	**regulation of cell cycle**	7.10E-06	0.011698	18	1.0824	297	1.8310
GO:0005876	**spindle microtubule**	8.70E-06	0.014002	13	0.7817	28	0.1726
GO:0006974	**response to DNA damage stimulus**	1.40E-05	0.022093	96	5.7727	349	2.1515
GO:0000226	**microtubule cytoskeleton organization and biogenesis**	2.56E-05	0.039427	11	0.6615	130	0.8014
GO:0006261<br>GO:0006262<br>GO:0006263	**DNA-dependent DNA replication**	2.78E-05	0.041836	16	0.9621	70	0.4315
GO:0006270	**DNA replication initiation**	2.87E-05	0.042237	12	0.7216	16	0.0986
GO:0006323	**DNA packaging**	4.85E-05	0.069897	1	0.0601	120	0.7398
GO:0043227	**membrane-bounded organelle**	5.27E-05	0.07292	824	49.5490	7588	46.7789
GO:0005643<br>GO:0005644	**nuclear pore**	5.27E-05	0.07292	24	1.4432	66	0.4069
GO:0051325	**interphase**	5.92E-05	0.079343	1	0.0601	93	0.5733
GO:0043231	**intracellular membrane-bounded organelle**	6.87E-05	0.089707	822	49.4287	7583	46.7480
GO:0000228	**nuclear chromosome**	7.10E-05	0.090988	7	0.4209	147	0.9062
GO:0006297	**nucleotide-excision repair, DNA gap filling**	7.58E-05	0.095362	12	0.7216	17	0.1048
GO:0000075	**cell cycle checkpoint**	8.14E-05	0.097099	6	0.3608	82	0.5055

numbers([a]), percentile([b]) and statistics([c]) of gene lists upregulated or downregulated([d]) after overexpression of *miR-195* among all genes([e]) involved in the GO term.
