# Supplementary material for: The Tumor-Suppressive miR-497-195 Cluster Targets Multiple Cell-Cycle Regulators in Hepatocellular Carcinoma
Source: PLoS One. 2013 Mar 27;8(3):e60155. doi: 10.1371/journal.pone.0060155 (PMC3609788; doi:10.1371/journal.pone.0060155)
Supplement: Table S1 — Primers for 3′UTR reporter assay. (ZIP) [file pone.0060155.s006.zip › Sup.tableS1-2.pdf]

Supplementary Table 1-2 Gene ontology analysis of genes, whose expression levels were changed by *miR-497* overexpression in Hep G2 (fold change > 2.0 in *miR-497*-overexpressed cells compared with *Luc*-overexpressed cells, 48 hours)

GO accession	GO term	*p*-value[c]	Corrected *p*-value[c]	Genes in selection[d]		Genes in total[e]	
				count[a]	%[b]	count[a]	%[b]
GO:0007049	**cell cycle**	6.82E-21	4.34E-16	183	17.1348	729	4.4942
GO:0022402	**cell cycle process**	1.71E-20	5.44E-16	95	8.8951	541	3.3352
GO:0022403	**cell cycle phase**	9.88E-20	2.09E-15	95	8.8951	397	2.4474
GO:0000279	**M phase**	1.40E-18	2.23E-14	95	8.8951	321	1.9789
GO:0007067	**mitosis**	4.10E-15	5.21E-11	77	7.2097	215	1.3254
GO:0000087	**M phase of mitotic cell cycle**	5.05E-15	5.35E-11	79	7.3970	219	1.3501
GO:0000793	**condensed chromosome**	9.98E-15	9.05E-11	34	3.1835	124	0.7644
GO:0000775	**chromosome, centromeric region**	1.65E-14	1.31E-10	48	4.4944	119	0.7336
GO:0000278	**mitotic cell cycle**	3.99E-14	2.82E-10	79	7.3970	355	2.1885
GO:0000779	**condensed chromosome, centromeric region**	2.23E-13	1.42E-09	27	2.5281	64	0.3946
GO:0051301	**cell division**	3.60E-13	2.08E-09	90	8.4270	279	1.7200
GO:0000777	**condensed chromosome kinetochore**	1.81E-11	9.59E-08	25	2.3408	57	0.3514
GO:0005694	**chromosome**	6.61E-11	3.23E-07	81	7.5843	433	2.6694
GO:0006260	**DNA replication**	1.51E-10	6.87E-07	57	5.3371	186	1.1467
GO:0007059	**chromosome segregation**	5.29E-10	2.24E-06	23	2.1536	80	0.4932
GO:0044427	**chromosomal part**	6.51E-10	2.58E-06	48	4.4944	358	2.2070
GO:0000776<br>GO:0005699	**kinetochore**	8.73E-10	3.26E-06	29	2.7154	75	0.4624
GO:0005819	**spindle**	1.95E-09	6.90E-06	43	4.0262	142	0.8754
GO:0006259	**DNA metabolic process**	3.56E-09	1.19E-05	61	5.7116	469	2.8913
GO:0015630	**microtubule cytoskeleton**	2.43E-07	7.01E-04	93	8.7079	516	3.1811
GO:0005856	**cytoskeleton**	2.43E-07	7.01E-04	179	16.7603	1260	7.7677
GO:0000819	**sister chromatid segregation**	2.32E-07	7.01E-04	7	0.6554	36	0.2219
GO:0044430	**cytoskeletal part**	2.85E-07	7.87E-04	91	8.5206	847	5.2216
GO:0000070<br>GO:0016359	**mitotic sister chromatid segregation**	7.15E-07	1.89E-03	6	0.5618	35	0.2158
GO:0007017	**microtubule-based process**	1.70E-06	4.33E-03	11	1.0300	250	1.5412
GO:0006950	**response to stress**	2.46E-06	6.01E-03	23	2.1536	1474	9.0870
GO:0007126	**meiosis**	9.38E-06	2.07E-02	17	1.5918	93	0.5733
GO:0051327	**M phase of meiotic cell cycle**	9.38E-06	2.07E-02	17	1.5918	93	0.5733
GO:0005876	**spindle microtubule**	9.43E-06	2.07E-02	12	1.1236	28	0.1726
GO:0000226	**microtubule cytoskeleton organization and biogenesis**	1.00E-05	2.12E-02	9	0.8427	130	0.8014
GO:0005874	**microtubule**	1.25E-05	0.024846	73	6.8352	254	1.5659
GO:0051321	**meiotic cell cycle**	1.24E-05	0.024846	17	1.5918	94	0.5795
GO:0005737	**cytoplasm**	2.10E-05	0.040492	835	78.1835	7017	43.2587
GO:0043228	**non-membrane-bounded organelle**	2.52E-05	0.045734	251	23.5019	2387	14.7155
GO:0043232	**intracellular non-membrane-bounded organelle**	2.52E-05	0.045734	251	23.5019	2387	14.7155
GO:0003824	**catalytic activity**	3.65E-05	0.064492	21	1.9663	4751	29.2892
GO:0022616	**DNA strand elongation**	5.00E-05	0.083515	1	0.0936	6	0.0370
GO:0051726<br>GO:0000074	**regulation of cell cycle**	4.89E-05	0.083515	10	0.9363	297	1.8310

numbers([a]), percentile([b]) and statistics([c]) of gene lists upregulated or downregulated([d]) after overexpression of *miR-497* among all genes([e]) involved in the GO term.
