# Supplementary material for: The Tumor-Suppressive miR-497-195 Cluster Targets Multiple Cell-Cycle Regulators in Hepatocellular Carcinoma
Source: PLoS One. 2013 Mar 27;8(3):e60155. doi: 10.1371/journal.pone.0060155 (PMC3609788; doi:10.1371/journal.pone.0060155)
Supplement: Table S1 — Primers for 3′UTR reporter assay. (ZIP) [file pone.0060155.s006.zip › Sup.tableS1-3.pdf]

Supplementary Table 1-3 Gene ontology analysis of genes, whose expression levels were changed by *miR-195* overexpression in sK-Hep-1 (fold change > 2.0 in *miR-195*-overexpressed cells compared with *Luc*-overexpressed cells, 48 hours)

GO accession	GO term	*p*-value[c]	Corrected *p*-value[c]	Genes in selection[d]		Genes in total[e]	
				count[a]	%[b]	count[a]	%[b]
GO:0022403	**cell cycle phase**	2.12E-30	1.50E-25	135	7.3932	397	2.4474
GO:0000279	**M phase**	5.59E-28	1.98E-23	134	7.3384	321	1.9789
GO:0022402	**cell cycle process**	3.00E-26	7.08E-22	136	7.4480	541	3.3352
GO:0007049	**cell cycle**	8.01E-26	1.42E-21	240	13.1435	729	4.4942
GO:0000087	**M phase of mitotic cell cycle**	1.66E-21	2.35E-17	108	5.9146	219	1.3501
GO:0007067	**mitosis**	2.80E-21	3.30E-17	105	5.7503	215	1.3254
GO:0000278	**mitotic cell cycle**	7.49E-19	7.57E-15	108	5.9146	355	2.1885
GO:0051301	**cell division**	7.58E-18	6.70E-14	122	6.6813	279	1.7200
GO:0006260	**DNA replication**	5.27E-16	3.73E-12	77	4.2169	186	1.1467
GO:0000793	**condensed chromosome**	4.75E-16	3.73E-12	47	2.5739	124	0.7644
GO:0005694	**chromosome**	1.38E-13	8.88E-10	119	6.5170	433	2.6694
GO:0007059	**chromosome segregation**	1.27E-12	7.50E-09	29	1.5882	80	0.4932
GO:0006259	**DNA metabolic process**	9.20E-12	5.01E-08	134	7.3384	469	2.8913
GO:0044427	**chromosomal part**	2.62E-11	1.26E-07	52	2.8478	358	2.2070
GO:0000775	**chromosome, centromeric region**	2.67E-11	1.26E-07	52	2.8478	119	0.7336
GO:0007017	**microtubule-based process**	4.01E-10	1.77E-06	62	3.3954	250	1.5412
GO:0000779	**condensed chromosome, centromeric region**	8.11E-10	3.37E-06	28	1.5334	64	0.3946
GO:0006950	**response to stress**	1.93E-09	7.57E-06	156	8.5433	1474	9.0870
GO:0000777	**condensed chromosome kinetochore**	3.76E-09	1.40E-05	27	1.4786	57	0.3514
GO:0007051	**spindle organization and biogenesis**	1.18E-08	4.18E-05	7	0.3834	45	0.2774
GO:0005819	**spindle**	1.50E-08	5.05E-05	39	2.1358	142	0.8754
GO:0003774	**motor activity**	1.81E-08	5.81E-05	56	3.0668	137	0.8446
GO:0003777	**microtubule motor activity**	1.90E-08	5.83E-05	40	2.1906	74	0.4562
GO:0000070<br>GO:0016359	**mitotic sister chromatid segregation**	2.33E-08	6.87E-05	9	0.4929	35	0.2158
GO:0006974	**response to DNA damage stimulus**	2.85E-08	8.06E-05	109	5.9693	349	2.1515
GO:0051321	**meiotic cell cycle**	5.39E-08	1.46E-04	25	1.3691	94	0.5795
GO:0000819	**sister chromatid segregation**	5.74E-08	1.50E-04	9	0.4929	36	0.2219
GO:0007010	**cytoskeleton organization and biogenesis**	6.43E-08	1.63E-04	83	4.5455	532	3.2797
GO:0007126	**meiosis**	1.10E-07	2.56E-04	25	1.3691	93	0.5733
GO:0051327	**M phase of meiotic cell cycle**	1.10E-07	2.56E-04	25	1.3691	93	0.5733
GO:0015630	**microtubule cytoskeleton**	1.12E-07	2.56E-04	108	5.9146	516	3.1811
GO:0000776<br>GO:0005699	**kinetochore**	1.17E-07	2.58E-04	32	1.7525	75	0.4624
GO:0000794	**condensed nuclear chromosome**	1.32E-07	2.83E-04	14	0.7667	46	0.2836
GO:0000226	**microtubule cytoskeleton organization and biogenesis**	2.03E-07	4.22E-04	15	0.8215	130	0.8014
GO:0002376	**immune system process**	2.84E-07	0.000574	22	1.2048	887	5.4682
GO:0000075	**cell cycle checkpoint**	8.78E-07	0.001725	7	0.3834	82	0.5055
GO:0005615	**extracellular space**	1.16E-06	0.002217	212	11.6101	655	4.0380
GO:0006261<br>GO:0006262<br>GO:0006263	**DNA-dependent DNA replication**	1.45E-06	0.00269	6	0.3286	70	0.4315
GO:0006281	**DNA repair**	1.53E-06	0.002767	78	4.2716	272	1.6768
GO:0006297	**nucleotide-excision repair, DNA gap filling**	1.86E-06	0.003212	14	0.7667	17	0.1048
GO:0005576	**extracellular region**	1.83E-06	0.003212	544	29.7919	1816	11.1954
GO:0000228	**nuclear chromosome**	2.72E-06	0.004577	22	1.2048	147	0.9062
GO:0030705	**cytoskeleton-dependent intracellular transport**	2.86E-06	0.004699	44	2.4096	124	0.7644
GO:0005874	**microtubule**	3.31E-06	0.005319	84	4.6002	254	1.5659
GO:0044421	**extracellular region part**	3.59E-06	0.005635	212	11.6101	914	5.6347
GO:0009605	**response to external stimulus**	3.98E-06	0.006124	19	1.0405	822	5.0675
GO:0051726<br>GO:0000074	**regulation of cell cycle**	4.90E-06	0.007371	21	1.1501	297	1.8310
GO:0051325	**interphase**	7.44E-06	0.010966	1	0.0548	93	0.5733
GO:0007154	**cell communication**	8.10E-06	0.011693	496	27.1632	3372	20.7879
GO:0050896<br>GO:0051869	**response to stimulus**	1.07E-05	0.015067	219	11.9934	2835	17.4773
GO:0044430	**cytoskeletal part**	1.26E-05	0.017408	104	5.6955	847	5.2216
GO:0048856	**anatomical structure development**	1.41E-05	0.019166	22	1.2048	2331	14.3703
GO:0042770	**DNA damage response, signal transduction**	2.07E-05	0.026614	1	0.0548	71	0.4377
GO:0048731	**system development**	2.11E-05	0.026614	22	1.2048	2101	12.9523
GO:0007165	**signal transduction**	2.10E-05	0.026614	477	26.1227	2991	18.4391
GO:0007018	**microtubule-based movement**	2.23E-05	0.027641	44	2.4096	102	0.6288
GO:0030554	**adenyl nucleotide binding**	3.85E-05	0.04455	427	23.3844	1507	9.2904
GO:0002520	**immune system development**	3.73E-05	0.04455	20	1.0953	251	1.5474
GO:0048534	**hemopoietic or lymphoid organ development**	3.66E-05	0.04455	20	1.0953	235	1.4487
GO:0007242	**intracellular signaling cascade**	3.83E-05	0.04455	84	4.6002	1264	7.7924
GO:0030097	**hemopoiesis**	4.65E-05	0.052203	20	1.0953	211	1.3008
GO:0009611<br>GO:0002245	**response to wounding**	5.61E-05	0.06194	16	0.8762	481	2.9653
GO:0030154	**cell differentiation**	6.00E-05	0.065334	134	7.3384	1456	8.9760
GO:0006996	**organelle organization and biogenesis**	6.74E-05	0.072236	100	5.4765	1343	8.2794
GO:0008283	**cell proliferation**	8.67E-05	0.091487	90	4.9288	354	2.1824
GO:0005524	**ATP binding**	9.23E-05	0.095999	427	23.3844	1412	8.7048

numbers([a]), percentile([b]) and statistics([c]) of gene lists upregulated or downregulated([d]) after overexpression of *miR-195* among all genes([e]) involved in the GO term.
