# Supplementary material for: The Tumor-Suppressive miR-497-195 Cluster Targets Multiple Cell-Cycle Regulators in Hepatocellular Carcinoma
Source: PLoS One. 2013 Mar 27;8(3):e60155. doi: 10.1371/journal.pone.0060155 (PMC3609788; doi:10.1371/journal.pone.0060155)
Supplement: Table S1 — Primers for 3′UTR reporter assay. (ZIP) [file pone.0060155.s006.zip › Sup.tableS1-4.pdf]

Supplementary Table 1-4 Gene ontology analysis of genes, whose expression levels were changed by *miR-497* overexpression in sK-Hep-1 (fold change > 2.0 in *miR-497*-overexpressed cells compared with *Luc*-overexpressed cells, 48 hours)

GO accession	GO term	*p*-value[c]	Corrected *p*-value[c]	Genes in selection[d]		Genes in total[e]	
				count[a]	%[b]	count[a]	%[b]
GO:0022403	cell cycle phase	1.62E-23	8.35E-19	77	11.9938	397	2.4474
GO:0000279	M phase	5.11E-22	1.32E-17	77	11.9938	321	1.9789
GO:0022402	cell cycle process	7.73E-21	9.96E-17	77	11.9938	541	3.3352
GO:0007049	cell cycle	6.08E-21	9.96E-17	130	20.2492	729	4.4942
GO:0000087	M phase of mitotic cell cycle	4.28E-19	4.41E-15	66	10.2804	219	1.3501
GO:0007067	mitosis	5.59E-19	4.80E-15	64	9.9688	215	1.3254
GO:0006950	response to stress	5.19E-18	3.82E-14	132	20.5607	1474	9.0870
GO:0000278	mitotic cell cycle	1.40E-17	9.04E-14	66	10.2804	355	2.1885
GO:0051301	cell division	5.75E-17	3.29E-13	68	10.5919	279	1.7200
GO:0000775	chromosome, centromeric region	2.83E-15	1.46E-11	36	5.6075	119	0.7336
GO:0000793	condensed chromosome	3.44E-15	1.61E-11	28	4.3614	124	0.7644
GO:0005694	chromosome	3.06E-14	1.31E-10	64	9.9688	433	2.6694
GO:0007059	chromosome segregation	4.68E-13	1.77E-09	26	4.0498	80	0.4932
GO:0000779	condensed chromosome, centromeric region	4.82E-13	1.77E-09	21	3.2710	64	0.3946
GO:0015630	microtubule cytoskeleton	7.37E-12	2.40E-08	91	14.1745	516	3.1811
GO:0044427	chromosomal part	7.44E-12	2.40E-08	39	6.0748	358	2.2070
GO:0000777	condensed chromosome kinetochore	3.75E-11	1.14E-07	20	3.1153	57	0.3514
GO:0005819	spindle	7.37E-11	2.11E-07	33	5.1402	142	0.8754
GO:0006974	response to DNA damage stimulus	8.07E-11	2.19E-07	60	9.3458	349	2.1515
GO:0006259	DNA metabolic process	8.03E-10	2.07E-06	63	9.8131	469	2.8913
GO:0050896<br>GO:0051869	response to stimulus	1.27E-09	3.12E-06	205	31.9315	2835	17.4773
GO:0007017	microtubule-based process	2.98E-09	6.98E-06	12	1.8692	250	1.5412
GO:0000776<br>GO:0005699	kinetochore	9.42E-09	2.06E-05	21	3.2710	75	0.4624
GO:0006260	DNA replication	9.61E-09	2.06E-05	35	5.4517	186	1.1467
GO:0006281	DNA repair	1.36E-08	2.80E-05	36	5.6075	272	1.6768
GO:0000070<br>GO:0016359	mitotic sister chromatid segregation	2.89E-08	5.72E-05	13	2.0249	35	0.2158
GO:0000226	microtubule cytoskeleton organization and biogenesis	3.00E-08	5.72E-05	10	1.5576	130	0.8014
GO:0000819	sister chromatid segregation	4.91E-08	9.04E-05	13	2.0249	36	0.2219
GO:0007051	spindle organization and biogenesis	7.62E-08	1.35E-04	5	0.7788	45	0.2774
GO:0044421	extracellular region part	1.09E-07	1.87E-04	105	16.3551	914	5.6347
GO:0000075	cell cycle checkpoint	3.30E-07	5.49E-04	9	1.4019	82	0.5055
GO:0005576	extracellular region	3.55E-07	5.72E-04	258	40.1869	1816	11.1954
GO:0009611<br>GO:0002245	response to wounding	4.36E-07	6.80E-04	51	7.9439	481	2.9653
GO:0005874	microtubule	5.24E-07	7.94E-04	51	7.9439	254	1.5659
GO:0044430	cytoskeletal part	8.82E-07	0.001298	89	13.8629	847	5.2216
GO:0005815	microtubule organizing center	1.04E-06	0.00149	40	6.2305	240	1.4796
GO:0009605	response to external stimulus	1.24E-06	0.001684	68	10.5919	822	5.0675
GO:0006954	inflammatory response	1.24E-06	0.001684	45	7.0093	300	1.8495
GO:0005813	centrosome	1.44E-06	0.001903	39	6.0748	217	1.3378
GO:0002376	immune system process	1.67E-06	0.0021	76	11.8380	887	5.4682
GO:0005615	extracellular space	1.65E-06	0.0021	105	16.3551	655	4.0380
GO:0005876	spindle microtubule	2.41E-06	0.002894	10	1.5576	28	0.1726
GO:0006952<br>GO:0002217<br>GO:0042829	defense response	2.40E-06	0.002894	52	8.0997	536	3.3044
GO:0030593	neutrophil chemotaxis	4.66E-06	0.005456	10	1.5576	18	0.1110
GO:0006955	immune response	5.25E-06	0.006006	66	10.2804	597	3.6804
GO:0042611	MHC protein complex	5.70E-06	0.006385	8	1.2461	34	0.2096
GO:0002526	acute inflammatory response	6.53E-06	0.007159	3	0.4673	89	0.5487
GO:0033554	cellular response to stress	7.96E-06	0.008542	7	1.0903	148	0.9124
GO:0051726<br>GO:0000074	regulation of cell cycle	8.33E-06	0.00876	16	2.4922	297	1.8310
GO:0009987<br>GO:0008151<br>GO:0050875	cellular process	9.69E-06	0.009985	285	44.3925	10275	63.3438
GO:0042127	regulation of cell proliferation	1.17E-05	0.011834	50	7.7882	728	4.4880
GO:0002544	chronic inflammatory response	1.57E-05	0.015192	3	0.4673	5	0.0308
GO:0042613	MHC class II protein complex	1.59E-05	0.015192	8	1.2461	13	0.0801
GO:0031570	DNA integrity checkpoint	1.62E-05	0.015192	7	1.0903	46	0.2836
GO:0008285	negative regulation of cell proliferation	1.62E-05	0.015192	46	7.1651	331	2.0406
GO:0051716	cellular response to stimulus	1.87E-05	0.017217	7	1.0903	224	1.3809
GO:0002504	antigen processing and presentation of peptide or polysaccharide antigen via MHC class II	2.39E-05	0.021633	8	1.2461	17	0.1048
GO:0031100	organ regeneration	2.75E-05	0.024402	11	1.7134	25	0.1541
GO:0003777	microtubule motor activity	2.96E-05	0.02543	20	3.1153	74	0.4562
GO:0007010	cytoskeleton organization and biogenesis	2.92E-05	0.02543	20	3.1153	532	3.2797
GO:0006935	chemotaxis	4.25E-05	0.034544	31	4.8287	153	0.9432
GO:0006826<br>GO:0015681	iron ion transport	4.29E-05	0.034544	10	1.5576	26	0.1603
GO:0042330	taxis	4.25E-05	0.034544	31	4.8287	153	0.9432
GO:0051276<br>GO:0007001<br>GO:0051277	chromosome organization and biogenesis	4.16E-05	0.034544	21	3.2710	456	2.8112
GO:0007126	meiosis	4.61E-05	0.03603	12	1.8692	93	0.5733
GO:0051327	M phase of meiotic cell cycle	4.61E-05	0.03603	12	1.8692	93	0.5733
GO:0051321	meiotic cell cycle	5.56E-05	0.042774	12	1.8692	94	0.5795
GO:0030595	leukocyte chemotaxis	6.41E-05	0.048609	11	1.7134	36	0.2219
GO:0000796<br>GO:0005676<br>GO:0008620	condensin complex	8.59E-05	0.063203	3	0.4673	6	0.0370
GO:0007076	mitotic chromosome condensation	9.03E-05	0.065551	7	1.0903	12	0.0740
GO:0000077	DNA damage checkpoint	9.29E-05	0.066491	7	1.0903	42	0.2589
GO:0001775	cell activation	1.13E-04	0.079537	2	0.3115	254	1.5659
GO:0042221	response to chemical stimulus	1.21E-04	0.084089	36	5.6075	1099	6.7752
GO:0006996	organelle organization and biogenesis	1.31E-04	0.090048	41	6.3863	1343	8.2794
GO:0007346	regulation of mitotic cell cycle	1.40E-04	0.093433	2	0.3115	149	0.9186
GO:0033089	positive regulation of T cell differentiation in the thymus	1.44E-04	0.095081	4	0.6231	4	0.0247

numbers([a]), percentile([b]) and statistics([c]) of gene lists upregulated or downregulated([d]) after overexpression of *miR-497* among all genes([e]) involved in the GO term.
