# Supplementary material for: The Tumor-Suppressive miR-497-195 Cluster Targets Multiple Cell-Cycle Regulators in Hepatocellular Carcinoma
Source: PLoS One. 2013 Mar 27;8(3):e60155. doi: 10.1371/journal.pone.0060155 (PMC3609788; doi:10.1371/journal.pone.0060155)
Supplement: Table S2 — GO analysis of genes, whose expression levels were changed (>2-fold change) by miR-195 or miR-497 overexpression compared to control Luc-overexpression in Hep G2 (S2-1 or S2-2) or sK-Hep-1 (S2-3 or S2-4) cells 48 hours after transfection. (PDF) [file pone.0060155.s007.pdf]

Supplementary Table S2 Top 20 genes ranked by the fold enrichment score in Ago2-IP-seq with miRNA target predictions

Rank	Gene Symbol	fold enrichment score vs non-treated cells	Criteria of target site prediction			
			3'UTR sequence[a]	microRNA.org	Microcosm	TargetScan
**Genes enriched in Ago2-IP fraction of *miR-195*-overexpressed Hep G2 cells**						
1	ARL2	47.837	matched	+	+	
2	PTCH2	40.300	matched			
3	CA2	32.255	matched	+	+	
4	TNFSF9	16.863	matched			
5	TUBA1A	15.890	matched	+	+	+
6	MCART6	15.081	matched	+	+	
7	XKR8	14.709	matched			
8	ERN2	14.665		+	+	
9	BIK	14.611	matched			
10	FSTL1	13.928	matched	+	+	
11	RPS14	12.932	matched			
12	RABAC1	12.747	matched			+
13	CCNE1	12.562	matched	+	+	
14	CBLC	12.318	matched			
15	CXorf40A	12.165	matched	+	+	
16	SNCG	12.097	matched	+	+	+
17	POF1B	11.723	matched	+	+	
18	IHH	11.494	matched	+	+	
19	OSCAR	10.997		+	+	
20	PPP1R11	10.936		+	+	
**Genes enriched in Ago2-IP fraction of *miR-497*-overexpressed Hep G2 cells**						
1	NOS2	23.734	matched	+	+	
2	POF1B	15.818	matched	+	+	
3	SCN2A	15.813	matched	+	+	
4	HNRNPH2	14.957		+	+	
5	RGPD4	14.501	matched	+	+	
6	RARB	14.282	matched	+	+	
7	RFX4	13.842		+	+	
8	PTCH2	11.754	matched			
9	FSTL1	9.887	matched	+	+	
10	TUBA1A	7.855	matched	+	+	+
11	SPDYE6	7.414		+	+	
12	C1orf114	6.594		+	+	
13	ZNF568	6.329		+	+	
14	FHAD1	5.936				
15	AQP4	5.934	matched			
16	DLX5	5.933				
17	PPFIA4	5.931		+	+	
18	TTC23L	5.538		+	+	
19	MAMDC2	5.274		+	+	
20	TMEM200A	5.086		+	+	

[a]Genes hoboring the *miR-195* and *miR-497* targeting sequence (5'-ACGACGA-3') in the 3'UTR of mRNA are shown as "matched"
