# Supplementary material for: The Tumor-Suppressive miR-497-195 Cluster Targets Multiple Cell-Cycle Regulators in Hepatocellular Carcinoma
Source: PLoS One. 2013 Mar 27;8(3):e60155. doi: 10.1371/journal.pone.0060155 (PMC3609788; doi:10.1371/journal.pone.0060155)
Supplement: Table S3 — Top 20 genes ranked by the fold enrichment score in Ago2-IP-seq with miRNA target predictions. (PDF) [file pone.0060155.s008.pdf]

**Supplemenatry Table S3   Significance of identified target genes for *miR-195* and *miR-497* in Ago2-IP-seq, expression array experiments, and statistical analysis**

| | Ago2-IP-seq in Hep G2 | | | | Expression array (log2 ratio vs control transfectants) | | | | Correlation of expression in HCC primary cases* | | | |
|---|---|---|---|---|---|---|---|---|---|---|---|---|
| | ***miR-195*** | | ***miR-497*** | | **Hep G2** | | **sK-Hep-1** | | ***miR-195*** | | ***miR-497*** | |
| **GeneName** | **fold enrichment** | **rank** | **fold enrichment** | **rank** | ***miR-195*** | ***miR-497*** | ***miR-195*** | ***miR-497*** | **correlation coefficient*** | ***p*-value** | **correlation coefficient*** | ***p*-value** |
| ***CDK6*** | 2.4518615 | 553 | **1.5193529** | 210 | -1.22087 | -1.32380 | -1.40482 | -1.41466 | **-0.2570845** | **0.02** | **-0.3091761** | **0.00** |
| ***CCND1*** | **4.0197213** | 184 | **1.0197937** | 915 | -0.05767 | -0.77935 | -1.56689 | -1.33998 | 0.2661775 | 0.01 | 0.2611633 | 0.01 |
| ***CCND3*** | **6.6434619** | 50 | 0.8435109 | 1451 | -2.97051 | -2.52965 | -3.06233 | -1.76106 | 0.0859502 | 0.42 | 0.0988667 | 0.36 |
| ***CCNE1*** | **12.5615966** | 14 | **2.3372704** | 78 | -1.71522 | -1.41478 | -1.94184 | -1.06548 | **-0.3586009** | **0.00** | **-0.2994528** | **0.00** |
| ***CDC25A*** | **5.1771055** | 97 | **0.9662803** | 1075 | -4.73985 | -3.18519 | -5.11893 | -2.39788 | **-0.3153836** | **0.00** | **-0.2454694** | **0.02** |
| ***E2F3*** | 2.0676892 | 785 | **1.1333364** | 595 | -0.25458 | -0.48169 | -0.60576 | -0.22030 | **-0.2677048** | **0.01** | -0.1685326 | 0.11 |
| ***CDK4*** | **2.9600333** | 359 | 0.7883319 | 1635 | -1.73786 | -0.95476 | -1.77535 | -0.80360 | **-0.3554960** | **0.00** | **-0.2923810** | **0.01** |
| ***BTRC*** | **2.9344809** | 371 | 1.3661312 | 309 | -2.11719 | -1.65169 | -1.12690 | -0.99834 | -0.1901542 | 0.07 | -0.1282887 | 0.23 |

*Pearson's product-moment correlation coefficient were calculated for *miR-195* or *miR-497* and each target genes from dataset of HCC primary cases (GSE20596)
