# Supplementary material for: The Tumor-Suppressive miR-497-195 Cluster Targets Multiple Cell-Cycle Regulators in Hepatocellular Carcinoma
Source: PLoS One. 2013 Mar 27;8(3):e60155. doi: 10.1371/journal.pone.0060155 (PMC3609788; doi:10.1371/journal.pone.0060155)
Supplement: Table S4 — Significance of identified candidate targets for miR-195 and miR-497 through Ago2-IP-seq, expression array experiments, and statistical analyses. (PDF) [file pone.0060155.s009.pdf]

Supplementary Table S4 Primers for 3'UTR reporter assays

Target gene	Region	Forward primer for 5' to 3'	Reverse primer for 5' to 3'	Product size
CCND1	F1	TTGAGCTCAGGCAGGCGGGCGCCA	TTGAGCTCTCCCTCCTCTCCGGAGCA	3,186 bp
	F2	TTACGCGTGTAATTCTTGTAATTTTTATTTAGGA	TTAAGCTTAGATACACAGAAGCGATGTGA	224 bp
	F3	TTACGCGTTCACATCGCTTCTGTGTATCT	TTAAGCTTGCTGTTTCACAATACCTCATG	222 bp
CDK6	F1	TTACGCGTGGCCTCAGCAGCCGCCT	TTAAGCTTACCAAATCAGGCCCGGCA	390 bp
	F2	TTACGCGTTCTAGATTAAAACAGGCTTAGT	TTAAGCTTGATGTGCTACTCATTTTGCT	214 bp
	F3	TTACGCGTGCCGGTGCCTGAAATTATGT	TTAGCTTTCGGAGAATTGTGTTGTACT	165 bp
E2F3	F1	TTACGCGTACAAAGTGGGTTAGTACTACA	TTAAGCTTACATATAAACACACTCCTAACA	212 bp
CDK4	F1	TTACGCGTGCAATGGAGTGGCTGCCA	AAGCTTATAAAGGTAGGGAAAGGGACA	239 bp
CCNE1	F1	TTGAGCTCCCATCCTTCTCCACCAAAGA	TTACGCGTCAGTATTATCTTTATTAAAAAATGGA	519 bp
	F2	TTGAGCTCCCATCCTTCTCCACCAAAGA	TTACGCGTACACCTGCGAGGAGAGCCA	340 bp
	F3	TTGAGCTCTGGCTCTCCTCGCAGGTGT	TTACGCGTCAGTATTATCTTTATTAAAAAATGGA	198 bp
CCND3	F1	TTACGCGTCCCTGGAGAGGCCCTCT	TTAAGCTTCCAAGAAGCCAAAGCCAGT	1,006 bp
	F2	TTACGCGTCTTCTAAAGGTGTTGTCCCT	TTAAGCTTCAGGGTTACCACCACTTGT	343 bp
	F3	TTACGCGTATTAATTGCTTTGAGCACAACT	TTAAGCTTCCAAGAAGCCAAAGCCAGT	368 bp
CDC25A	F1	TTACGCGTCGGCAGGACCAGCCAGCA	TTAAGCTTGGGTTCAAGATCTTTTATTTTCA	1,762 bp
	F2	TTACGCGTGTCAAGCTGCTCTGAGCCA	TTAAGCTTTTGATGAAGTTGAATAAATTAAACA	196 bp
	F3	TTACGCGTACCTGTTATAAGTTATCGGACA	TTAAGCTTCTCCTATACCAGCCAATGTCA	244 bp
BTRC	F1	TTGAGCTCCACTCTGTCTACGGACCCT	TTACGCGTCACACTCCATTATCATGATCCT	4,314 bp
	F2	TTGAGCTCGACCTCATACTTGCCCAGGA	TTACGCGTTGGCCAAGTAGAAAACATTTCT	1,966 bp
	F3	TTGAGCTCAGAAATGTTTTCTACTTGGCCA	TTACGCGTGAAAGCTCTCGGGGAAGCT	2,065 bp
