# Supplementary material for: The Tumor-Suppressive miR-497-195 Cluster Targets Multiple Cell-Cycle Regulators in Hepatocellular Carcinoma
Source: PLoS One. 2013 Mar 27;8(3):e60155. doi: 10.1371/journal.pone.0060155 (PMC3609788; doi:10.1371/journal.pone.0060155)
Supplement: Table S5 — Primer sets for q- gPCR, promoter assay, bisulfite sequencing, and ChIP-PCR. (PDF) [file pone.0060155.s010.pdf]

Supplementary Table S5 Primers for Genomic real-time PCR, Promoter assay, Bisulfite sequencing, and ChIP-PCR

Assay name	Region	Forward primer for 5' to 3'	Reverse primer for 5' to 3'	Product size
**Genomic realtime PCR**	***miR-497***	TCCCTGGTTCCTCCCAAA	TGAAAGGAAGAGAGCACATAGCA	**121 bp**
	miR-195	TGTAAGCACCCCTCTAAAATCTCC	TGGGCAACAAAGACTCCACTT	**108 bp**
**Promoter assay**	**F1**	TTACGCGTAGCTAAGGGATAATCATAAGGT	TTAAGCTTCTCCCCTGAGATCCTGGT	**1,332 bp**
	**F2**	TTACGCGTACTGGGAGTGTGGGTGAGA	TTAAGCTTCTCCCCTGAGATCCTGGT	**899 bp**
	**F3**	TTACGCGTACCAGGATCTCAGGGGAGA	TTAAGCTTGAAAGGAAGAGAGCACATAG	**1,381 bp**
	**F4**	TTACGCGTCTATGTGCTCTCTTCCTTTCA	TTAAGCTTACCACCCTGCCTGGAGCA	**430 bp**
**Bisulfite sequence**	**1**	GTTTAGGATTTGAAGGGAGAAA	TTTAAAAATCACTCCCTCCTTC	**719 bp**
	**2**	GAAGGAGGGAGTGATTTTTAAA	TCTCCCCTAAAATCCTAATCC	**503 bp**
	**3**	TTTAGGGGAGAGTAGTTGAGGT	CTCAAAAAACAAAATCCAAAAA	**583 bp**
	**4**	GTTTTGGTTTTTGGATTTTGTT	AAAAACCAACACACTACCTCCT	**554 bp**
	**5**	TAGGAGGTAGTGTGTTGGTTTT	AAATACTTACATACCCCACCCT	**509 bp**
**ChIP-PCR**	**R1**	CAACAGCAATGGCAGATTCC	AAAGCCCGTTCCTCCCTTC	**360 bp**
	**R2**	AAATTGGCATCGGGACAGAG	TCCTGGACTAGCTCCCCAAA	**343 bp**
