# Supplementary material for: The Tumor-Suppressive miR-497-195 Cluster Targets Multiple Cell-Cycle Regulators in Hepatocellular Carcinoma
Source: PLoS One. 2013 Mar 27;8(3):e60155. doi: 10.1371/journal.pone.0060155 (PMC3609788; doi:10.1371/journal.pone.0060155)
Supplement: Methods S1 — (DOC) [file pone.0060155.s011.doc]

**Supplementary Methods S1**

**Genomic copy-number analysis by real-time genomic PCR**

Genomic copy-numbers of the region around *miR-195* and *miR-497* were assessed by SYBR Green q-gPCR with the KAPA SYBR FAST Universal 2×qPCR master mix (Kapa Biosystems) on an ABI Prism 7900 Sequence Detection System (Applied Biosystems). DNAs from one normal liver (C20) and six normal lymphoblastoid cell lines (CONT1-6) were used as controls. *COL7A1*, mapped to 3q21, was selected as a control for single copy genes . The primer pairs used for *miR-195* and *miR-497* are listed in Supplementary Table S5.

All results were analyzed on a standard curve derived from known concentrations of DNA samples. DNA from six normal lymphoblastoid cell lines was included in each assay, and the mean value was used to normalize the data and correct for inter-assay variation. The copy-numbers around *miR-195* and *miR-497* were calculated by dividing those values by the *COL7A1* values as described elsewhere . Values around <0.5 in ratio of controls were considered to represent heterozygous loss.

**Promoter assay**

Genomic DNA fragments of interest were obtained by PCR (Supplementary Table S5) and ligated into the vector pGL3-Basic (Promega, Madison, WI). An equal amount of each construct or an empty pGL3-Basic vector was introduced into cells along with an internal control vector (pRL-hTK, Promega) using Lipofectamine 2000 (Invitrogen, Carlsbad, CA) according to the manufacturer’s instructions. Firefly luciferase and *Renilla* luciferase activities were measured 48 hours after transfection using the Dual-Luciferase Reporter Assay System (Promega), and relative luciferase activity was calculated and normalized versus *Renilla* luciferase activity.

**Bisulfite sequencing**

Genomic DNA was treated with sodium bisulfite using an EZ DNA Methylation Kit^TM^ (Zymo Research, Orange, CA) according to the manufacturer’s instructions and subjected to a polymerase chain reaction (PCR) using primer sets designed to amplify regions of interest (Supplementary Table S5). 　For the bisulfite sequencing analysis, the PCR products were subcloned and then sequenced.

**Chromatin immunoprecipitation (ChIP) assay**

Cells were plated into 15-cm dishes at a density of 10^7^ cells per dish the day before cell fixation. After 24 hours, cells were fixed with 37% formaldehyde for 5 min, and ChIP assays were carried out using a ChIP-IT Express Enzymatic kit (Active Motif, Carlsbad, CA) and anti-H3K27me3, anti-H3K4me3 and anti-H3 antibodies according to the manufacturer’s instructions. Concentrations of ChIP-enriched DNA samples were normalized and subjected to qRT-PCR analysis with KAPA SYBR FAST Universal 2×qPCR master mix (Kapa Biosystems, Woburn, MA) on an ABI Prism 7900 Sequence Detection System (Applied Biosystems). Fold enrichment values by ChIP were assessed for H3K27me3 and H3K4me3 relative to H3 in genomic regions R1 and R2 (Supplementary Table S5).

**Gene Ontology (GO) analysis**

Fold changes of expression of each gene in cells 48 hours after transfection with miCENTURY OX miNatural mimicking *miR-195* or *miR-497* compared with that in control dsRNA-transfected counterparts were calculated by GeneSpring GX software version 11.5.1 (Agilent Technologies). Using the GeneSpring Gene Ontology Analysis feature, the significantly downregulated/upregulated (>2.0-fold) genes by *miR-195* or *miR-497* transfection were categorized into specific biological processes defined by the GeneSpring Gene Ontology Database. Each process, which contains significant genes, was ranked in significance, using a *p*-value overlap of experimentally significant genes with known genes in each biological process.

**Bioinformatic analysis for miRNA target identification**

Gene Set Enrichment Analysis (GSEA, www.broad.mit.edu/gsea) was performed with a false discovery rate (FDR) of 1% using gene lists of Ago2-IP candidates and gene expression profiling data changing after miRNA overexpression to statistically confirm the enrichment of Ago2-IP candidates in the group of genes downregulated by miRNA overexpression among total genes by the notion that target genes of miRNA should be downregulated by miRNA overexpression. Then pathway analyses were conducted on gene intersection of Ago2-IP candidates and downregulated genes after miRNA overexpression (>2.0-fold) using an Ingenuity Pathway Analysis (IPA, www.ingenity.com).

**Antibodies**

Antibodies used for western blotting were anti-cyclin D1 (CCND1; Cell Signaling Technology, Beverly,MA), anti-cyclin D3 (CCND3; Proteintech Group, Chicago, IL), anti-cyclin E1(CCND3; Proteintech Group), anti-cyclin-dependent kinase 6 (CDK6; Cell Signaling Technology), anti-cyclin-dependent kinase 4 (CDK4; Cell Signaling Technology), anti-E2F transcription factor 3 (E2F3; Upstate,), anti-cell division cycle 25 homolog A (CDC25A; Cell signaling), anti-beta-transducin repeat containing E3 ubiquitin protein ligase (BTRC; Cell signaling), and anti-beta-actin (Sigma).

**Supporting References**

1. Kozaki K, Imoto I, Pimkhaokham A, Hasegawa S, Tsuda H, Omura K, Inazawa J. PIK3CA mutation is an oncogenic aberration at advanced stages of oral squamous cell carcinoma. Cancer Sci. 2006;97:1351-1358.
